# Molecular evolution of NASP and conserved histone H3/H4 transport pathway

**DOI:** 10.1186/1471-2148-14-139

**Published:** 2014-06-20

**Authors:** Syed Nabeel-Shah, Kanwal Ashraf, Ronald E Pearlman, Jeffrey Fillingham

**Affiliations:** 1 Department of Chemistry and Biology, Ryerson University, 350 Victoria St., Toronto M5B 2K3, Canada; 2 Department of Biology, York University, 4700 Keele St., Toronto M3J 1P3, Canada

**Keywords:** NASP, H3/H4 transport, Hif1, N1/N2, Molecular evolution, Chromatin, Phylogenetics, SHNi-TPR, Histone chaperone

## Abstract

**Background:**

NASP is an essential protein in mammals that functions in histone transport pathways and maintenance of a soluble reservoir of histones H3/H4. NASP has been studied exclusively in Opisthokonta lineages where some functional diversity has been reported. In humans, growing evidence implicates NASP miss-regulation in the development of a variety of cancers. Although a comprehensive phylogenetic analysis is lacking, NASP-family proteins that possess four TPR motifs are thought to be widely distributed across eukaryotes.

**Results:**

We characterize the molecular evolution of NASP by systematically identifying putative NASP orthologs across diverse eukaryotic lineages ranging from excavata to those of the crown group. We detect extensive silent divergence at the nucleotide level suggesting the presence of strong purifying selection acting at the protein level. We also observe a selection bias for high frequencies of acidic residues which we hypothesize is a consequence of their critical function(s), further indicating the role of functional constraints operating on NASP evolution. Our data indicate that TPR1 and TPR4 constitute the most rapidly evolving functional units of NASP and may account for the functional diversity observed among well characterized family members. We also show that NASP paralogs in ray-finned fish have different genomic environments with clear differences in their GC content and have undergone significant changes at the protein level suggesting functional diversification.

**Conclusion:**

We draw four main conclusions from this study. First, wide distribution of NASP throughout eukaryotes suggests that it was likely present in the last eukaryotic common ancestor (LECA) possibly as an important innovation in the transport of H3/H4. Second, strong purifying selection operating at the protein level has influenced the nucleotide composition of NASP genes. Further, we show that selection has acted to maintain a high frequency of functionally relevant acidic amino acids in the region that interrupts TPR2. Third, functional diversity reported among several well characterized NASP family members can be explained in terms of quickly evolving TPR1 and TPR4 motifs. Fourth, NASP fish specific paralogs have significantly diverged at the protein level with NASP2 acquiring a NNR domain.

## Background

The fundamental repeating unit of eukaryotic chromatin is the nucleosome that wraps a 146 bp stretch of DNA around a histone octamer consisting of two of each of histone H2A, H2B, H3 and H4 [1]. The transport of histones from the cytoplasm to the nucleus and their subsequent assembly into nucleosomes is mediated by a diverse set of proteins including histone chaperones [2] that are classified into several families based on their binding specificities and sequence and structural similarities [2]. One group of histone H3/H4 chaperones is the **n**uclear **a**utoantigenic **s**perm **p**rotein (NASP) family also known as the N1/N2 family.

The founding member of the NASP family is *Xenopus laevis* N1/N2, which is expressed in oocytes and specifically binds histones H3/H4, providing a mechanism for the storage of the soluble histones required for DNA replication in the early embryo [3,4]. NASP is the mammalian homolog of N1/N2 and was first described in rabbit testes as a highly autoantigenic protein which shares greater than 50% similarity to N1/N2 [5,6]. In mammals, NASP predominantly exists as two alternatively spliced isoforms; one is considerably longer than the other and is expressed in embryonic tissues and testis (tNASP) whereas the smaller version which lacks a region of 339 amino acids is called the somatic NASP (sNASP) and is highly expressed in all dividing cells [7]. NASP expression is tightly cell cycle regulated and its over-expression causes delay in cell cycle progression at the G1/S border [7,8]. NASP expression is essential in mammals as its gene disruption results in early embryonic lethality in mice [9]. Previous studies have shown that human NASP co-purifies with replication dependent and independent histones H3.1 and H3.3 respectively [10,11]. In human cells, newly synthesized histones H3.1/H4 are thought to successively pass through at least four distinct cytosolic complexes [10,12]. In this context, NASP has been shown to be involved in accepting the histones from heat shock proteins, in the Hat1-dependent acetylation of H4, and subsequently handing over these histones to another histone chaperone, anti-silencing factor 1 (Asf1) through a physical interaction that has been shown to exist in humans and *Saccharomyces cerevisiae*[10,12,13].

NASP family proteins share conserved motifs, possessing four tetratricopeptide repeats (TPR) where the second TPR is typically interrupted by a large acidic domain [14]. The NASP structural organisation is conserved from fungi to mice forming the SHNi-TPR protein family that was named for three initially characterized members, *Silencing in the middle of the centromere protein 3* (**S**im3– *Schizosaccharomyces pombe*), ***H*** *at1p-interacting factor-1* (Hif1, *S.cerevisiae*), and ***N*** *ASP*-**i**nterrupted **TPR** repeats [14]. The TPR motifs are 34 amino acid long amphipathic helices that form a helix-turn-helix arrangement and are thought to provide a structural scaffold for mediating protein-protein interactions [15]. Different TPR motifs in human NASP show different binding affinities for either histone H3/H4 or H1. For example, the acidic patch present within TPR2 is critical for H1-binding whereas TPR4 mediates the interaction with core H3/H4 histones [16]. These studies suggest that NASP might be involved in multiple functions involving histone dynamics (for review see [17]).

In addition to mammals, NASP homologs have been detected in several eukaryotic models such as *S. cerevisiae* (Hif1), *S. pombe* (Sim3) and *Caenorhabditis elegans* (NASP-1) [14,18,19]. In addition to their conserved motif arrangement, these proteins share functional similarity in that they generally demonstrate histone H3/H4 chaperone activity [17]. Despite this, some functional diversity does exist among NASP family proteins. For example, Hif1 physically interacts with Hat1 and Hat2 as part of the Hat1 complex that functions in the acetylation of newly synthesized histone H4 [19,20], whereas Sim3 appears to specifically function in deposition of the centromeric H3 variant [14], and does not appear to physically interact with Hat1 [21,22]. In addition to this, a recent report suggests that Sim3 also has a general role in chromatin maintenance and acts as an H3/H4 chaperone with some overlapping functional characteristics with Asf1 [23]. In *C. elegans*, NASP-1 has been implicated in female development through its interactions with histone deacetylase and TRA-4 proteins [18]. Additionally, human NASP functions in the fine tuning of a soluble reservoir of histones H3/H4 by handing over excess histone H3 and H4 to heat shock proteins (HSP90/HSC70) for chaperone mediated autophagy [24].

In humans, NASP expression is significantly altered in a variety of cancers including those of the ovary and prostate [25-27]. Despite the demonstrated role of NASP in a wide range of cellular processes, questions remain about the underlying mechanistic details of NASP function. Recently, we found that a NASP family protein, NASP-related protein 1 (Nrp1) co-purifies with Asf1 in the ciliate protozoan *Tetrahymena thermophila*[28] suggesting that the Asf1-NASP physical interaction is evolutionarily conserved in eukaryotes. Molecular evolutionary analysis has the potential to provide useful insights into protein function as well as providing information about changes in interacting partners [29,30]. Molecular evolutionary analyses of the proteins involved in the transport of histones H3/H4 including HSP90, Asf1 and Importinβ have previously been reported and suggest that these proteins are highly conserved throughout the eukaryotes [31-33]. Although NASP has been suggested to be equally widely present among eukaryotes [34], a comprehensive phylogenetic analysis of NASP family proteins is lacking.

We present here a comprehensive phylogenetic analysis of NASP family proteins*.* Our analysis indicates that NASP is conserved across all of the major eukaryotic lineages ranging from the excavata to the crown group (animals, fungi, amoebozoans and plants) suggesting that the NASP histone chaperone was most likely present in the LECA. Furthermore, we show that in addition to the conserved arrangement of the four TPR motifs, an overall negatively charged nature is preserved in NASP family members suggesting that diversification of these proteins during eukaryotic evolution must have been determined by strong functional and structural constraints. High acidic amino acid composition in the TPR2 interruption region has been maintained by the presence of strong selection which in turn has biased the nucleotide composition in the corresponding *NASP* gene. Furthermore, we show that among ray-finned fish, NASP has undergone gene duplication and two paralogs exist under different genomic G + C environments which suggests functional diversification of the two proteins. The present work combined with previous evolutionary analyses on Asf1, HSP90 and Importinβ proteins, suggests that histones H3/H4 are transported via an evolutionarily conserved pathway that most likely was present in the last eukaryotic ancestor.

## Results

### NASP is highly conserved in eukaryotes

We used PSI-BLAST searches with default parameters to identify putative NASP orthologs across a wide range of eukaryotic lineages including those of the crown group (animals, fungi, amoebozoans and plants), the *Guillardia* nucleomorph, chromalveolates (apicomplexans, ciliates, oomycetes, and diatoms), and excavatas (parabasalids and kinetoplastida). Searches were carried out using the non-redundant database available at the NCBI website (http://www.ncbi.nlm.nih.gov/) and identified sequences were recovered in the reciprocal searches (for accession numbers see Additional file 1: Table S1). The NASP family proteins contain a Pfam domain (PF10516) called SHNi-TPR which is an interrupted form of the TPR motif [14]. In order to correctly identify putative NASP orthologs, all sequences we obtained were analysed using the Pfam database [35] to confirm the presence of SHNi-TPR (PF10516) (see Methods for details). We queried the eggNOG orthology database [36] using human NASP and *T. thermophila* Nrp1protein sequences as reference, and observed that NASP forms an orthology group (KOG4563) with 117 protein members from 90 different eukaryotic species including several that were identified through our PSI-BLAST searches. However, several previously unreported putative NASP orthologs not present in the eggNOG database were also identified in this study through PSI-BLAST searches.

The distinctive feature of NASP family proteins is the presence of a TPR motif which is interrupted by a large acidic tract [14], and also revealed by our comparative sequence alignments (see below). In order to gauge the broad distribution of NASP proteins among eukaryotes, we prepared a hidden Markov Model (HMM) profile of the alignment of this region of the sequences culled from PSI-BLAST searches and screened through UniProtKB using HMMsearch [37]. This resulted in the recovery of significant hits (e ≤ 10^-5^) throughout eukaryotes with the notable exception of diplomonads (Additional file 2). Recovered sequences were analysed for the motif organisation and presence of SHNi-TPR/PF10516. Thus NASP appears widely distributed across the eukaryotes and may have emerged very early during the eukaryotic diversification. In fact the identification of putative NASP proteins through BLAST searches in excavata lineages (e.g., Parabasalids (*Trichomonas vaginalis*) and kinetoplastids (*Trypanosoma*)) which are thought to be highly divergent eukaryotic lineages [38-40] suggest that NASP was likely present in the LECA. Despite our repeated searches, we were unable to identify any putative NASP orthologs in diplomonads suggesting loss of this gene in these lineages.

Synteny, or gene neighbourhood analysis, often provides a good indication of correct identification of orthologs within closely related species [41]. We analysed the synteny of NASP among sequenced tetrapods and some other vertebrates using Genomicus [42]. We observed that gene pairs *CCDC17*, *GPBP1L1* (coiled-coil domain containing 17 and GC-rich promoter binding protein 1-like 1, respectively) and *AKR1A1*, *PRDX1* (aldo-ketoreductase family 1, member A1 and peroxiredoxin 1, respectively) are found on either side of the *NASP* gene. We were able to trace back this conserved gene neighbourhood to the coelacanth *Latimeria chalumnae* which is considered the closest living relative of tetrapods [43]. These results suggest that the *NASP* genomic organisation is conserved among tetrapods.

### Conserved TPR domain organisation

NASP proteins are defined by the presence of four TPR motifs where the second TPR is typically interrupted by large acidic patches. In addition, the TPR4 in *S. pombe* has also been predicted to contain a small insertion of 5 residues within the linker region that connects the two helices [14]. Our multiple sequence alignment (MSA) analysis indicates that the TPR motif sequence and arrangement are well conserved with TPR1 and TPR3/4 flanking the interrupted TPR2 (Additional file 3: Figure S1A-E). The second residue of each TPR repeat often has a side chain that is either negatively charged or amidated [14]. For kinetoplastid lineages, we observed that the region interrupting TPR2 is significantly smaller than the others including humans, fungi and ciliates, consisting of 16 amino acid residues that mostly are hydrophobic and not predominantly acidic (For TPR organisation see: Additional file 3: Figure S1E). These kinetoplastid NASP do however possess an overall net negative charge with several acidic residues located mostly at the N-terminus. Furthermore, our analysis also indicates that *T. vaginalis* putative NASP is highly divergent and the TPR2 interruption region consists of only 10 residues with at least half of them acidic. However, several acidic residues are also found dispersed throughout the protein, thus giving it an overall net negative charge (data not shown). Interestingly, MSA and Pfam domain analysis also revealed that among a few selected lineages NASP has gained additional domains. For example, in addition to the conserved four TPRs, the putative NASP in *Trichoplax adhaerens* (phylum: Placozoa) contains an N-terminal MADF domain (PF10545) whereas *Phytophthora infestans* and *P. sojae* (both oomycetes) have gained an N-terminal Longin domain (PF13774). Such isolated instances of domain gain likely represent lineage specific functional divergence.

MSA analysis of NASP family TPRs from a wide range of eukaryotic lineages permitted us to identify highly conserved residues that have been kept almost unchanged throughout evolution. For example, we noted that in addition to conserved hydrophobic residues found in all four TPRs, the amino acids at positions 7 and 8 are also highly conserved (Additional file 3: Figure S1 A-E). For TPR1, the amino acids at positions 12 (leucine) and 21 (valine) have also remained almost unchanged suggesting that these residues are functionally relevant. Similarly, residues at positions 12 (leucine), 15 (alanine), 21 (tryptophan) and 24 (leucine) in TPR2 as well as positions 9 (glutamic acid), 11 (serine), 28/30 (leucine/leucine or isoleucine) in TPR3 and positions 10 (alanine) and 31 (isoleucine or leucine) of TPR4 are highly conserved. In addition, selected positions contain amino acids with similar biochemical properties (see the MSA in Additional file 3). It is also worth noting that a previously reported small C-terminal basic patch that could function as a nuclear localization signal [17] is also conserved in all NASP family members studied here (data not shown). Nevertheless, we also observed some lineage specific variations at selected amino acid positions. For instance, among vertebrates, TPR1 amino acid positions 6 and 9 invariably are glycine and glutamine respectively, whereas among insects these residues have been substituted with serine and lysine, respectively. Similarly, amino acid positions 9 and 6 (both lysine) in TPR2 and TPR3, respectively, are highly conserved among vertebrates whereas they are more variable among other lineages (see the MSA in Additional file 3). The wide distribution of NASP in eukaryotic super-groups as well as the conserved pattern of TPR motifs strongly suggests that these amino acid variations represent a case of functional divergence among different orthologous proteins. In accordance with Dunleavy et al. [14] we also detected small insertions in the TPR4 of certain fungal lineages including *S. pombe*, *S. japonicus*, *Cryptococcus gattii*, *Candida albicans*, *Coccidioides immitis* and *Neurospora crassa.* Furthermore, *Albugo laibachii* (a genus of oomycetes) and *Thalassiosira pseudonana* (marine centric diatom) have each also experienced an insertion of 38 residues in the TPR4 (Additional file 3: Figure S1-D). The functional importance of these insertions is unknown. They may however represent convergent changes that arose independently among different lineages.

### Phylogenetic analysis of NASP

**Figure 1:**
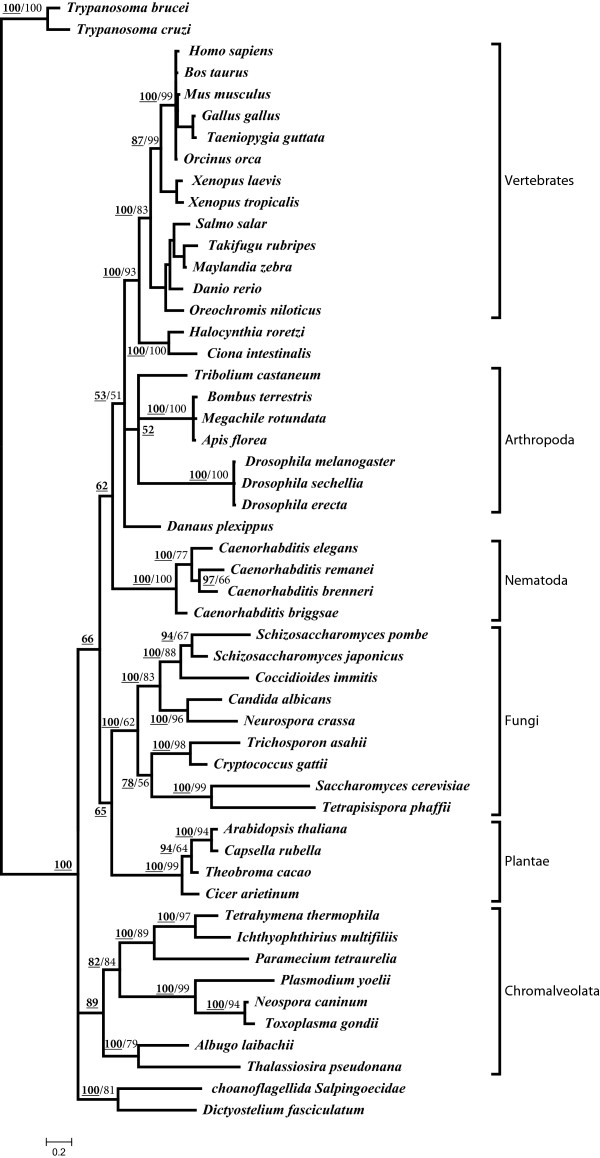
**Phylogenetic tree of NASP proteins from different eukaryotic lineages reconstructed using TPR 1–4 amino acid sequences.** Tree topology and branch lengths correspond to Bayesian inferences. The average standard deviation of split frequencies from two runs was 0.009. Posterior probability values are indicated in bold-face and underlined whereas bootstrap values (based on 1000 replicates) for the ML tree are indicated in light-face and are only reported when at least ≥ 50%. Different taxonomic groups are indicated in the right margin. *T. cruzi* and *T. brucei* were used as out groups to root the tree.

After preliminary alignment and phylogenetic analyses, we reduced the total number of sequences representing different eukaryotic super groups for further investigation. Our global sequence alignments (data not shown) indicate that the TPRs constitute the most conserved regions of NASP family proteins. We therefore used the conserved blocks of amino acids comprising TPRs1-4 to reconstruct the protein phylogeny. The phylogeny shown in Figure 1 was reconstructed using MSA of 52 putative NASP protein sequences identified across diverse eukaryotic lineages. In order to assess the robustness of our results, we combined Bayesian analysis and maximum likelihood methods under the rtREV + F substitution model with gamma distributions as predicted by Prottest version 3.2 [44] and MEGAv5.2.1 [45]. *Trypanosoma cruzi* and *T. brucei* were used as out groups since these organisms are believed to diverge earlier than any other organism represented on the tree (Figure 1) [46-49].

The resulting phylogenetic tree recovered different taxonomic groups with well-defined topology as indicated by high statistical support for each internal node (Figure 1). This highly structured tree topology closely resembles the eukaryotic phylogeny inferred from rRNA sequences [46,49]. Thus with a few notable exceptions (see below), the overall topology suggests that NASP evolution parallels eukaryotic evolution. Chromalveolates branched first forming a monophyletic group followed by the diversification of the crown group lineages. Significant diversification is observed among different chromalveolata lineages as evidenced from the average amino acid variations (*p* = 0.721 ± 0.019 substitutions per site) along with the varying branch lengths and grouping patterns on the tree (Figure 1). Ciliates form their own subgroup which is closely related to the apicomplexans with a monophyletic origin whereas oomycetes and diatoms are more closely related to each other than to the ciliates or apicomplexans. The crown group lineages with the exception of choanoflagellates and amoebozoans form a monophyletic group with a dichotomous topology such that fungi and plants fall within one monophyletic group whereas the remaining animals fall within another group.

The NASP differentiation within fungal and plant lineages occurred at the same time as evidenced from the monophyly of these groups with a moderate statistical support (ML bootstrap support of 35, Bayesian posterior probability of 65; Figure 1). This pattern of divergence deviates from accepted eukaryotic species phylogeny in which plants and fungi acquire distinct monophyletic groups [46-49]. Figure 1 demonstrates that plant lineages cluster together with relatively short branch lengths suggesting that NASP is highly conserved within plants. This observation is further supported by estimating average evolutionary divergence values (*p* = 0.251±0.027 substitutions per site) for plant lineages. Fungal species form their own monophyletic subgroup with significant diversity apparent within basidiomycota and most ascomycota lineages. Long branches and moderately high average of amino acid sequence variations (*p* = 0.696±0.019 substitutions per site) among nine fungal lineages provide further support for the presence of considerable differentiation among these proteins. Interestingly, *S. cerevisiae* and another related ascomycota fungus *Tetrapisispora phaffii*[50] appear to be closely related to each other and quite divergent from the remaining ascomycotes as can be seen from their position in the phylogeny which is closer to basidiomycotes (*Trichosporon asahii* and *C. gattii*) than other ascomycota lineages. This suggests that Hif1 of *S. cerevisiae* and *T. phaffii* might have evolved faster in comparison to the other fungal lineages.

The chordate lineages form a monophyletic sub-group within which tunicates, which are thought to be the closest living relatives of vertebrates [51], group together and take the basal position followed by diversification among vertebrate lineages. Further analysis of the tree topology reveals that NASP lineages corresponding to fish cluster together whereas the differentiation of NASP in amphibians is followed by its diversification within mammals and aves. For NASP lineages corresponding to mammals and aves, we observed a minor disagreement between ML and Bayesian analyses. For the ML tree, aves lineages form a distinct subgroup as a sister clade to the mammals. Under Bayesian analysis however the differentiation of mammals and aves appears to have occurred concurrently. This discrepancy is consistent with the relatively low statistical support for the ML tree (Figure 1). As noted earlier, choanoflagellates and amoebozoans are the two exceptions to the general trend of NASP evolution seen for the crown group lineages. Both of these lineages take the basal position in the phylogeny before the differentiation of chromalveolates indicating that these proteins are highly diverged and may be subject to strict lineage-specific functional constraints. Consistent with this we observed a long stretch of serine/threonine tandem repeats at the N-terminus of amoebozoan lineages (data not shown). Human NASP proteins have previously been predicted to contain a large number of potential serine/threonine phosphorylation sites with a few found towards the protein’s N-terminus [17]. However, the presence of serine/threonine tandem repeats at the N-terminus may represent a lineage-specific adaptation although the functional significance remains unclear. An examination of multiple sequence alignments (Additional file 3: Figure S1, A-D) indicates amino acid variations at certain highly conserved and structurally relevant positions in TPRs1-4 for choanoflagellates. For example, glycine is invariably found at position 8 of TPR1 (Additional file 3). However in choanoflagellates, it has been substituted to the basic residue lysine. Furthermore, position 17 of TPR1 and position 4 of TPR2 in choanoflagellates have been substituted by lysine and glutamine respectively in place of conserved hydrophobic residues (Additional file 3: Figure S1-A,B). Similarly, we observed that position 9 of TPR3 which almost invariably contains an acidic amino acid (glutamic acid/aspartic acid) has been replaced by a serine which is a polar neutral amino acid and only becomes acidic when phosphorylated, and highly conserved hydrophobic positions 21 and 13 have been substituted by glutamic acid and glutamine residues for TPR3 and TPR4, respectively (Additional file 3: Figure S1-C, D). Such variation at apparently key amino acid positions in the functional units (TPRs in this case) of a protein could possibly impact/alter its function and/or interactions with other proteins.

To further examine evolutionary relationships among NASP proteins, we reconstructed a protein phylogeny based on complete protein sequences. The resulting phylogeny (Additional file 4: Figure S2) recovered the tree topology virtually identical to the one presented in Figure 1. The low statistical support for the tree, and slightly larger distances are indicative of an overall low similarity of the entire protein sequences (compare Table 1 with Additional file 1: Table S2). Nevertheless, this phylogeny based on complete protein sequences does provide two useful insights. First, the phylogenetic tree shown in Figure 1 is the most likely representation of NASP evolution with minor topological differences observed between the two phylogenies accounted for by the low statistical support for the phylogeny based on the entire protein sequence (Additional file 4: Figure S2; Figure 1). Second, NASP evolution is essentially dictated by the constraints present on TPR motifs.

**Table 1:** **Average number of amino acid and nucleotide variations along with average synonymous (*p***_**S**_**) and non-synonymous (*p***_**N**_**) differences per site among NASP lineages from various taxonomic groups**

	***p***_**AA**_**(SE)**	***p***_**NT**_**(SE)**	***p***_**S**_**(SE)**	***p***_**N**_**(SE)**	***R***^***a***^	**Z-test**^**b**^
Vertebrate	0.430±0.016	0.353±0.008	0.617±0.010	0.263±0.011	0.98	21.928^***^
Tunicata	0.602±0.023	0.417±0.012	0.715±0.032	0.323±0.019	0.87	10.599^***^
Arthropoda	0.561±0.012	0.435±0.008	0.611±0.012	0.375±0.011	1.06	11.673^***^
Nematoda	0.451±0.020	0.365±0.010	0.676±0.020	0.265±0.014	1.05	16.227^***^
Fungi	0.726±0.014	0.578±0.008	0.765±0.010	0.522±0.011	0.56	14.94^***^
Plants	0.484±0.017	0.400±0.013	0.620±0.027	0.327±0.017	0.8	8.617^***^
Ciliates	0.689±0.019	0.465±0.011	0.631±0.024	0.426±0.015	0.6	7.19^***^
Apicomplex	0.531±0.020	0.401±0.011	0.655±0.021	0.315±0.015	0.9	12.447^***^
Euglenozoa	0.423±0.024	0.367±0.014	0.703±0.030	0.256±0.016	0.87	12.856^***^

*p*_AA,_*p*_NT,_*p*_S,_ and *p*_N,_ represent average number of amino acid, nucleotide, synonymous and non-synonymous nucleotide differences per site when calculated using the entire protein/nucleotide coding sequence along with the Z-test of selection. SE indicates standard error based on 1000 bootstrap replicates.

^a^average transition/transversion ratio.

^b^H_1_: *p*_*N*_<*p*_*S*_ and H_o_: *p*_*N =*_*p*_*S*_.

^***^ P< 0.001.

TPR motifs provide a platform to mediate protein-protein interactions and distinct NASP TPRs have been shown to have differential affinity for histones H1 and H3/H4 [15]. By estimating the divergence among different regions of the protein, specifically the TPRs (TPR1-4 combined in this case), the acidic region and the remaining N- and C-termini (Additional file 1: Table S2), we observed significantly lower amino acid variation for the TPRs in comparison to the other regions, indicating that the TPRs constitute the most conserved regions in NASP proteins. Table 2 summarizes the overall amino acid variations (*p*-distances) for individual TPRs (TPR 1–4) across all the lineages without distinguishing among different taxonomic groups. From the estimated average amino acid *p*-distances, it appears that the TPR1 and TPR4 regions might be diverging more rapidly than TPR 2 and TPR3 (Table 2). This observation is further supported by calculating the amino acid *p*-distances for individual TPRs discriminating among different taxonomic groups which indicates that TPR1 and TPR4 generally have higher amino acid variations relative to TPR2 and TPR3 (Additional file 1: Table S3). In order to derive additional insight into the evolutionary processes that are shaping NASP TPRs, we re-constructed separate phylogenies for each TPR motif (Additional file 5: Figure S3, A-D). Difference in the clustering patterns among individual TPRs and/or divergence from the main protein phylogeny (Figure 1) has the potential to indicate how similar/dissimilar functional constraints might be present among different lineages for a given TPR. However when individual TPRs are studied in isolation, the reduction in total number of amino acid sites lowers the statistical support for the resulting trees, diminishing the significance of any useful inferences. Nevertheless, based on average *p*-distance values shown in Table 2 and Additional file 1: Table S3, it appears that TPR1 and TPR4 are the fastest evolving functional units of a given NASP ortholog and as such may account for the functional diversity observed among well characterized NASP family members.

**Table 2:** Average number of amino acid and nucleotide variations among different TPR domains

	***p***_**AA**_**(SE)**	***p***_**NT**_**(SE)**	***p***_**S**_**(SE)**	***p***_**N**_**(SE)**	***R***^***a***^	**Z-test**^**b**^
TPR1	0.764±0.026	0.599±0.015	0.688±0.009	0.568±0.022	0.9^#^	4.602^***^
TPR2	0.617±0.036	0.499±0.021	0.665±0.016	0.437±0.027	1^#^	7.098^***^
TPR3	0.682±0.034	0.530±0.022	0.687±0.013	0.476±0.031	0.88^#^	5.646^***^
TPR4	0.762±0.028	0.582±0.016	0.633±0.015	0.564±0.023	1.2^#^	2.105^**^
Entire Protein	0.729±0.012	0.562±0.008	0.725±0.006	0.510±0.011	0.64	16.823^***^

*p*_AA,_*p*_NT,_*p*_S,_ and *p*_N,_ represent the average number of amino acid, nucleotide, synonymous and non-synonymous nucleotide differences per site along with the Z-test of selection. SE indicates standard error based on 1000 bootstrap replicates.

^a^average transition/transversion ratio.

^#^calculated using Maximum composite likelihood method in this case.

^b^H_1_:*p*_*N*_<*p*_*S*_ and H_o_:*p*_*N =*_*p*_*S*_.

^***^P< 0.001; ^**^P<0.05.

### Constraints on NASP evolution

We employed several complementary methods to estimate the selective forces operating on NASP evolution. We used the NASP nucleotide coding sequences from the representative lineages of all eukaryotic super groups (Additional file 6: figure S4-A). We carried out a codon-based Z-test of selection by comparing synonymous and non-synonymousvariations. Our results reveal extensive synonymous variation, considerably higher than non-synonymous variations (***P<0.001) in all comparisons (Table 1). In fact the level of silent substitutions was generally very high when compared across all the species (*p*_S_(SE)=0.73±0.006, *p*_N_(SE)=0.5± 0.01; ***P<0.001; Additional file 6: Figure S4-A). These results suggest the presence of strong purifying selection acting at the protein level, presumably to maintain appropriate structure required for NASP function. To ascertain the functional constraints operating on different regions of the protein, we extended our analysis by distinguishing between TPRs (combined), the acidic region and the remaining parts of the protein for different taxonomic groups (Additional file 1: Table S2), as well as among individual TPRs (Table 2). The results indicate that the amount of silent variation is always significantly higher than the non-silent variation in all Z-test comparisons (for *P-*values and test statistics refer to Table 2 and Additional file 1: Table S2). Table 2 also reveals that although the number of synonymous variations is always higher than the non-synonymous, the non-synonymous variations are significantly greater for TPR1/4 in comparison to TPR2/3. These results support our observation that TPR1/4 are diverging more quickly than the TPR2/3. In addition, overall nucleotide diversity for four TPRs calculated using a sliding window approach indicates that TPR1 and TPR4 exhibit higher amounts of divergence relative to TPR2/3 (Additional file 6: Figure S4-B).

Positive selection often affects only a few sites during a protein’s molecular evolution. We therefore conducted an ML-based analysis for detecting the nature of selective forces operating upon each individual codon in *NASP* coding sequences among different taxonomic groups. We used the program HyPhy to conduct the analysis (see Methods for details) [52]. Our results do not indicate that any codons have been under positive selection at the statistical threshold of P<0.05 (codon data not shown). These studies suggest that the purifying selection has been the dominant factor during NASP evolution.

We assessed the codon usage bias among different NASP lineages. It has been shown that different organisms exhibit preferences for the use of different synonymous codons [53]. The strength of codon bias has been shown to vary across genes within each genome and factors influencing codon bias may include selection for translational accuracy and efficiency, and GC bias [54-56]. We calculated the overall degree of codon bias for NASP genes among different taxonomic groups using the effective number of codons (ENC) [57]. The higher ENC value of 61 signifies that all synonymous codons are equally used and there is no bias in codon usage whereas a lower value of 20 indicates an extreme bias suggesting that only a preferred codon is used in each synonymous class. In the case of NASP we did not find any significant codon bias with the exception of ciliates (Table 3). The overall ENC values range from 49.89± 5.82 (for tunicates) to 54.64± 3.43 (for insects) whereas ciliates indicate a slightly biased trend with ENC of 40.73± 2.37 in comparison to the other chromalveolata lineages (**P< 0.05, t-test). Ciliates are known to have AT rich genomes and have previously been reported to exhibit a strong bias toward codons with low GC content [58,59] (Additional file 7: Figure S5). The observed lack of any significant codon usage bias among *NASP* genes is possibly due to the strong functional constraints at the protein level that allow for an extensive amount of silent divergence which in turn results in a decrease in codon usage bias. However, highly conserved and functionally relevant sites often exhibit a stronger bias in codon usage [60,61]. This possibility awaits further analysis for NASP among different lineages. Our analysis however of relative synonymous codon usage (RSCU) on human NASP suggests that highly conserved TPR residues isoleucine and arginine are preferentially encoded by AUU and AGA, respectively (for detailed view see: Additional file 7: Figure S6).

**Table 3:** Codon usage bias referred to as the effective number of codons (ENC) estimated in NASP discriminating different taxonomic groups

**Taxonomic group**	**ENC**
Vertebrate	50.69±1.41
Tunicate	49.89±2.58
Arthropod	54.64±3.43
Nematode	51.13±2.59
Fungi	50.98±5.82
Plant	53.99±2.82
Ciliate	40.73±2.37
Apicomplexa	53.18±5.97
Euglenozoa	53.78±2.63

**Figure 2:**
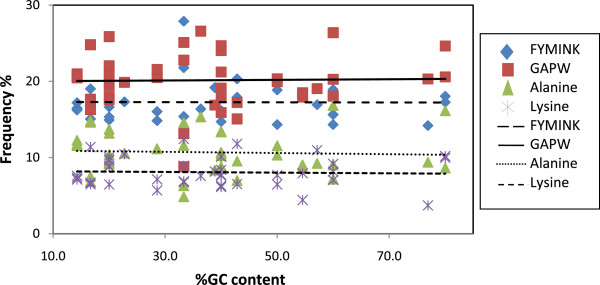
**Relationship between the genomic GC content and GC-rich (GAPW) and GC-poor (FYMINK) residues.** The relationship between most represented residues in each class (Alanine and Lysine) and GC content is also shown.

We investigated the role of natural selection for certain biased amino acids by determining the correlation coefficients between the genomic GC content and the frequency of GC-rich (GAPW) and GC poor (FYMINK) amino acids. Under the neutral model of evolution, GC rich and GC poor residues should positively and negatively correlate with the genomic GC content, respectively [62]. However, there will be no correlation if selection has influenced their frequency. Our results indicate the absence of any significant correlation between the genomic GC content and the frequency of GC-rich residues and GC-poor residues (Figure 2; Table 4). Alanine (GC rich) and lysine (GC poor) represent the most abundant residues in each class. We do not observe significant correlation between the genomic GC content and the most represented amino acids in each class (alanine and lysine) (Figure 2, Table 4). These results are not consistent with the neutral model expectations and suggest a role for natural selection to maintain high frequencies of certain amino acids.

**Table 4:** Genomic GC correlation with GC-rich and GC-poor amino acids in NASP family proteins

**NASP family**	**Spearman rank correlation coefficient (*r***_***S***_**)**	**P-value**
Genomic GC vs. GAPW	-0.0896	0.899
Genomic GC vs. FYMINL	0.0634	0.968
Genomic GC vs. Alanine	-0.188	0.749
Genomic GC vs. Lysine	0.0405	0.796

To further assess the role of natural selection, we compared the changes at the first codon position (non-synonymous) with those at the third codon position (synonymous) in the most abundant residues. The nucleotide frequencies at these two codon positions should not be significantly different from each other according to the expectations of the neutral model [62]. Codons for the GC-rich residue alanine possess G at the first position whereas codons for the GC-poor residue lysine have A at the first position. Our analysis indicates that the mean G+A content at the first position (69.7±3.8) is significantly greater than the mean G+A content (46.54±15.8) at the four fold degenerate sites (*t* test = 8.98, *p= 0.000*). Furthermore, the mean G+A content at the first codon position and at four-fold degenerate sites do not significantly correlate (*r*_S_= 0.01, *p*-value*=* 0.945). These results suggest that selection has influenced the nucleotide composition to maintain a high frequency of alanine and lysine.

Glutamic acid and aspartic acid are also found from high to moderate frequencies as determined by the amino acid composition of NASP family proteins (data not shown; Additional file 1: Table S4). Codons for both of these residues have G at their first position as do alanine codons. It is therefore reasonable to assume that the elevated levels of G+A at the first codon position might be due to the selection for glutamic and aspartic acids in addition to alanine and lysine. This conclusion is further supported by the observation that nucleotide differences between the first and the third codon positions for alanine and lysine was most evident in the acidic domain that interrupts the TPR2 of the NASP proteins. The mean G+A content at the first position (81.0±7.6) is significantly greater than the mean G+A content (6.7±23.1) at the four fold degenerate sites (*t* test = 20.05, *p= 0.000*). Furthermore, Z-test of selection also indicates the presence of strong purifying selection on the acidic domain of NASP proteins (Additional file 1: Table S2).

Provided that the clustering pattern of negatively charged NASP proteins is well differentiated with respect to different taxonomic groups (Figure 1), we assessed the potential role in selection of electrostatic interaction properties of different NASP proteins from each eukaryotic representative group (Additional file 7: Figure S7). Given that the crystal structure is not at present available for any of the NASP proteins, we used the I-TASSER server for structural predictions [63]. Subsequently, the predicted structures were used to estimate the electrostatic potentials and their similarity indices using the web-PIPSA pipeline [64]. Our results indicate that differences in electrostatic potential is not the major evolutionary driving force as the representative NASP proteins from different taxonomic groups do not follow any particular clustering patterns (Additional file 7: Figure S7). These results suggest that selection has continuously acted in one direction to maintain high amounts of negatively charged amino acids particularly in the acidic domain, resulting in an overall net negative charge of NASP proteins presumably for their interaction with positively charged histones (also see Additional file 1: Table S4).

### NASP duplication

In addition to the NASP splice variants present in most vertebrate species which generate functional diversity in NASP (for review see [17]), we observed that selected lineages have undergone gene duplication events. *Gorilla, Felis* and *Taeniopygia* among chordates and *Caenorhabditis* among nematodes each contain two NASP paralogs. Furthermore, we observed that among chordates, the ray-finned fish lineages also have undergone the gene duplication in the ancestral genome of clupeocephala (~320 million years ago) [65], and *Danio rerio* has subsequently lost one copy of the gene. In most cases each of the two NASP paralogs has protein coding splice variants as assessed by ‘ensembl’ gene annotation [66]. This may generate additional functional diversity although the significance remains obscure.

**Figure 3:**
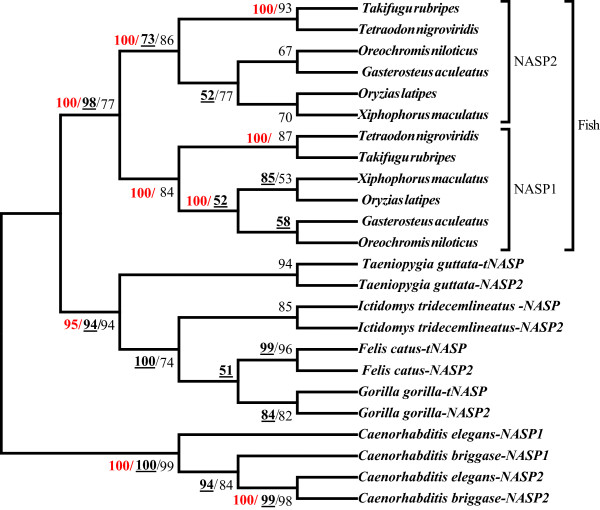
**Phylogenetic relationship among different NASP paralogs inferred by ML, MP and Bayesian methods.** The tree topology corresponds to ML estimations under the Tamura-Nei model. Branch lengths do not reflect genetic distance. Duplicated genes are referred to as NASP1 and NASP2 (see text for details). Confidence values for ML and MP trees are based on 1000 bootstrap replicates for each method and are indicated (≥50%) in light-face and bold-face (underlined), respectively. Bayesian posterior probability values are indicated in red (≥50%).

To further assess the relationship among NASP paralogs, we reconstructed a phylogenetic tree using the nucleotide coding sequences from the above lineages by ML, maximum-parsimony (MP) and Bayesian methods (Figure 3). Our analysis indicates that NASP paralogs exhibit a polyphyletic origin and different NASP subtypes cluster based on the species to which they belong rather than the NASP subtypes (Figure 3). However, in the case of the fish lineages the clustering pattern is based on NASP subtypes rather than species indicating that the duplication event has only occurred once. Similarly, *Caenorhabditis* lineages also display a clustering pattern based on type rather than species suggesting that the gene duplication event occurred prior to the diversification of various *Caenorhabditis* species. Based on the phyletic patterns (Figure 3) and following the nomenclature for NASP paralogs already established for *Caenorhabditis* lineages, we designated the two paralogs as *NASP1* and *NASP2* for each of the lineage specific duplicated genes.

### Different genomic contexts for NASP1 and NASP2

To gain insight into the genomic environment in which the two paralogs reside we analysed the synteny of duplicated genes. We observed that similar to other tetrapods, the ancestral *NASP* in the genome of clupeocephala has *CCDC17* and *AKR1A1* as its neighbours. Furthermore, among ray-finned fish lineages *GPBP1L1* and *PRDX1* genes which are also found in the *NASP* neighbourhood among other tetrapods are present as the immediate neighbours for at least one of the NASP paralogs (*NASP1*) whereas the direct neighbours of the *NASP2* include *GPC5* and *LRRC7* genes. Along with *NASP*, the *TMEM69* gene which is found in the *NASP* neighbourhood among most of the vertebrates has also been duplicated suggesting a large segmental duplication event. This duplication (~320MYA) may represent the signature of the whole genome duplication that took place in the ancestor of ray-finned fish [67]. Among other vertebrate lineage specific paralogs, *NASP1* has the conserved gene neighbourhood (see above) whereas *NASP2* neighbours include *CHD7* in *Gorilla, BICD1* in *Felis catus* and a gene that encodes a putative Zinc Finger protein in *Taeniopygia guttata.* Our analysis also indicates that the *NASP1* immediate neighbour in *C. elegans* is *APC-17*, whereas an uncharacterized gene lies next to the *NASP2*. From the analysis of gene structure and predicted protein sequences for *T. guttata* and *F. catus* paralogs, we observed that NASP2 are relatively smaller proteins (105 and 152 amino acids, respectively) and comprise only N- and C-termini respectively of the NASP1in these lineages. As these proteins lack TPR motifs or any other known functional units of NASP, we omitted them from further analyses.

**Figure 4:**
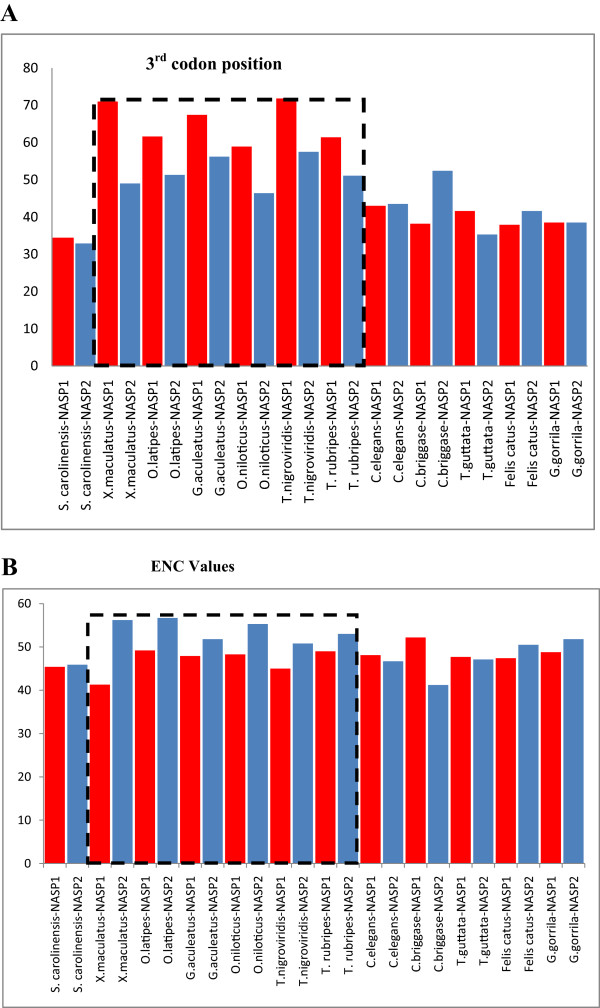
**Comparison of %GC3 and ENC values among NASP paralogs. A-** GC content at the third codon position of NASP paralogs among different lineages. The box signifies estimated values for %GC3 in fish lineages. **B**- The extent of codon usage bias referred to as ENC values among two NASP paralogs. The box indicates ENC for fish lineages.

Immediately after a hypothetical gene duplication event, purifying selection is expected to be relatively relaxed due to an initial functional redundancy, and to some extent it may permit the accumulation of divergence between the two paralogs [68-70]. To explain this in terms of protein function, two possible scenarios have been suggested: 1) in the case of sub-functionalization, the ancestral protein’s function(s) are distributed between the duplicated genes; 2) in the case of neo-functionalization, the duplicated protein acquires a new function(s) [71,72]. To gain insight into possible functional divergence, we assessed genomic G+C composition of the two paralogs. GC content has been correlated with various genomic properties, such as methylation patterns, recombination rates, gene density and gene expression patterns [73-76]. In addition, low GC content has also been associated with late replication [77] whereas highly expressed genes tend to be GC-rich [78]. In order to assess how evolutionary forces might have impacted on the genomic GC properties of the two paralogs, we calculated the GC content at the third codon position (GC3) because it has been shown to provide a good estimation of the GC content of the region in which the gene is located [79,80]. We observed a compositional bias in the GC content at the third codon positions for the fishes and *Caenorhabditis* whereas other lineage specific NASP paralogs did not show any significant differences in their %GC3 (Figure 4A). We found that *NASP1* always has a higher GC content than *NASP2* among ray-finned fish whereas the opposite is true in the case of *Caenorhabditis* lineages*.* The most significant difference was noted for the fish *Xiphophorus maculatus* in which the two paralogs have 71% and 49% GC content at the third codon positions for *NASP1* and *NASP2*, respectively (Figure 4A). Among fish lineages, *NASP1* and *NASP2* neighbouring genes *GPBP1L1*/*PRDX1* and *GPC5*/*LRRC7*, respectively, also showed similar GC3 divergence. *NASP1* neighbours (*GPBP1L1*/*PRDX1*) generally have a higher %GC3 (overall average: 70%/64%, respectively) in comparison to the *NASP2* neighbours (*GPC5*/*LRRC7*; overall average: 58%/60%, respectively) (Additional file 8: Figure S8 B-C). These observations suggest that the compositional bias among fish paralogs most likely arose after the duplication event due to their differential genomic locations. In contrast to the fishes the *Caenorhabditis NASP*1/2 neighbouring genes did not indicate any significant GC3 variations.

The single *NASP* in the distantly related *D. rerio* clusters with *NASP2* on the phylogenetic tree (Additional file 8: FigureS8A), and has a 42.5% GC3 (Additional file 7: Figure S5), suggesting that the lost copy of the gene is *NASP1*. However, the syntenic organisation of *D. rerio NASP* resembles that of *NASP1* where it directly neighbours *CCDC17* and *AKR1A1* whereas *GPBP1L1* is found next to *CCDC17.* Low %GC3 for *D. rerio* NASP correlates with the previous findings that the *D. rerio* genome has a lower GC content relative to the other fishes e.g., *Oryzias latipes* and *Tetraodon*[81].

The differences in the genomic environments may have a role in acquiring paralog-specific functions through distinct mutational biases [82] (for review, see [83]). High GC content has previously been correlated with elevated levels of gene transcription [84] as well as with the extent of codon usage bias [85]. We assessed the extent of codon bias between the two paralogs and observed that among fish lineages, *NASP1* demonstrates more biased trends in codon usage than the *NASP2* (Figure 4B). Similar trends were also seen for their corresponding neighbouring genes (Additional file 8: Figure S8 B-C). Furthermore, it is also evident from Figure 4B that among *Caenorhabditis*, *NASP2* exhibits slightly more biased codon usage than *NASP1*. Codon usage bias has previously been linked to selection for translational efficiency required for highly expressed genes and it has been shown that the preferred codons are often those which are recognised by the most frequent tRNAs [86]. For ray-finned fish and *Caenorhabditis* specific *NASP* paralogs however, compositional bias (G+C content) appears to be an important factor in shaping the codon usage. The observed compositional and codon usage bias could be linked to differential gene expression which has been proposed to provide indirect evidence for sub-functionalization and/or neo-functionalization [87].

### Functional differentiation of NASP Paralogs

**Figure 5:**
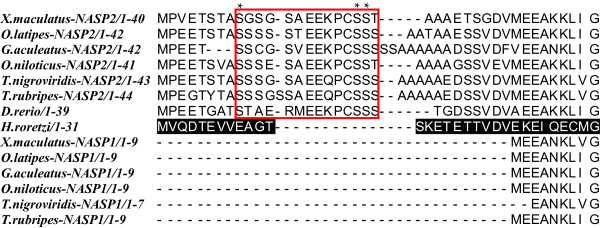
**Multiple sequence alignments of the N-terminal region for NASP paralogs found in different fish lineages.** The NNR region is highlighted in box whereas the out group lineage of *Halocynthia roretzi* is indicated in black. The asterisks (*) indicate the predicted conserved phosphorylation sites.

We examined the amino acid variations between the two paralogs in order to gain insights into functional differentiation. We observed that among fish lineages NASP1 has lost the N-terminal region consisting of the first 25–30 amino acid residues that are found upstream of the start of TPR1. We also detected a small region within these N-terminal residues that is highly conserved in NASP2 for all sequenced fish lineages (Figure 5). We refer to this conserved region as the NNR domain (***N*** ASP ***N***-terminal ***R*** egion) and it demonstrates a characteristic pattern of S-X(5)-E-E-X-P-C-S-S-(S/T) where X is any amino acid. The significance of this domain is currently unclear. However it carries potential serine phosphorylation sites, as predicted by the KinasePhos 2.0 server [88], which may allow functionally important post-translational modifications considering their conserved nature in fish NASP2 (Figure 5). The single retained copy of NASP in *D. rerio* also contains this NNR domain (Figure 5), further emphasizing our observations that the retained copy of the gene is *NASP2* in this lineage.

We analysed the four TPR motifs and found that NASP paralogs in fish and *Caenorhabditis* have maintained the conserved arrangement of four TPRs whereas *Gorilla* NASP2 has lost the entire TPR1 and partially the TPR2 (see below). We focused on fish-specific NASP1/2 due to their clear pattern of evolutionary divergence as evident from Figure 3. We compared the two paralogous proteins with the reconstructed ancestral sequence and observed amino acid substitutions at key residues within each TPR suggesting that both lineages have diverged from the ancestral state (For MSA Additional file 8: Figure S8D-G). Our analysis indicates that TPR4 accumulated the greatest number of variations with 15 residues substituted followed by TPR1which has 6 residues changed. In order to assess the selective pressures operating on different regions of the two paralogs, we conducted a codon based Z-test of selection for each pair of sequences. While our analysis suggests the presence of purifying selection (data not shown and Additional file 1: Table S5), it should be noted that after a gene duplication, positive selection may affect only a few residues that might not be detected by pair-wise comparison methods [89]. Nevertheless amino acid variations in the functional units along with the loss of N-terminal residues from NASP1 and the presence of an NNR domain in NASP2 provides indirect evidence for the functional differentiation between the two paralogs.

In contrast to the fish lineages, *Gorilla* NASP2 has lost the entire TPR1 and part of TPR2, as noted above. Furthermore, in the TPR2 interruption region it also lacks a stretch of 141 amino acid residues that are present in the NASP1. As a result NASP2 (582 amino acids in length) is much smaller than NASP1 (789 amino acids). Ensemble gene annotation indicates that unlike its other mammalian homologs (e.g. humans), *Gorilla* NASP1 does not have any splice variants equivalent in size to sNASP. These observations suggest that NASP2 might have assumed important functions regarding histone dynamics, e.g., similar to sNASP in other mammals. Our MSA analysis shows that the remaining regions of two paralogs including TPRs 3/4 are highly conserved with only a few amino acid substitutions (Additional file 8: Figure S8H-K). Low level of amino acid substitutions suggest that gene duplication probably occurred relatively recently. A Z-test of selection indicates that while most regions of the two proteins are evolving neutrally, the TPR2 interruption region demonstrates signs of positive selection (Additional file 1: Table S6). These results suggest that despite overall sequence conservation between *Gorilla* NASP1 and NASP2, the TPR2 interruption region has diverged significantly under the positive selection, possibly acquiring new function(s).

### NASP interacting partners are conserved

NASP functions in the H3/H4 transport pathway and interacts with a histone chaperone Asf1 as well as an Impβ which is a karyopherin-β family protein [10,12,13]. To estimate the wide distribution of NASP interacting proteins, we used Asf1 and Impβ protein sequences from well characterized family members such as humans and *S. cerevisiae* and explored the non-redundant database with PSI-BLAST using default parameters. Reciprocally recovered sequences for both Asf1 and Impβ were used to prepare an HMMprofile for each of the proteins and Hmmsearch was conducted to scan the UniProKb. We recovered the putative Asf1 and Impβ orthologs throughout the major eukaryotes, consistent with previous reports [31,33] (See Additional file 9 and Additional file 10 for accession numbers and species). The karyopherin-β family proteins contain a Pfam domain IBN_N (PF03810) whereas the Asf1 family has a PF04729 domain. The recovered sequences from Hmmsearch were analysed for the presence of these domains in each case. The presence of Asf1 and Impβ proteins throughout the major eukaryotic groups suggests that similar to NASP, these proteins are also highly conserved and likely were present in the LECA.

## Discussion

NASP is an essential protein in mammals and has a critical role in the trafficking of newly synthesized histones H3/H4 [10,12]. However, until now there has been a lack of information regarding the molecular evolution of NASP. We carried out a detailed phylogenetic analysis and establish that NASP evolution parallels that of eukaryotes. We also shed light on the selective forces operating on the evolution of NASP.

### Evolutionary conservation of NASP

Our ability to detect NASP in a wide range of eukaryotic lineages including highly divergent *T. vaginalis* and an organism with a reduced genome (*G. theta*), argues that the protein is widely distributed throughout eukaryotes, and is a key generalized histone chaperone that most likely was present in the LECA. The histone chaperone Asf1 which has been shown to physically interact with NASP in humans, budding yeast and the ciliate protozoan *T. thermophila*[10,13,28] is also highly conserved throughout the eukaryotes [32], and most likely was present in the LECA. We propose that that the NASP-Asf1-Importinβ interaction is equally ancient and has a critical role in histone H3/H4 metabolism.

Despite our extensive searches, we were unable to detect putative NASP homologs in diplomonads. This apparent loss is analogous to the absence of Histone Periodic Control 2 (Hpc2) in diplomonads [32]. Hpc2 is a subunit of an evolutionarily conserved H3/H4 chaperone Histone Regulator (HIR) complex which physically interacts with Asf1 during replication independent chromatin assembly [90]. The HUN domain of Hpc2 has been suggested to be a histone tail-binding subunit of the HIR complex which might be critical for the transfer of H3/H4 from Asf1 [32] and has been shown to be crucial for the HIR complex stability and preferential deposition of H3.3 [91].

Protein families are often categorized by the presence or absence of a conserved motif. In the case of NASP, the possession of interrupted forms of TPR (SHNi-TPR) motifs defines this family [14]. We have extended previous findings and shown that the structural arrangement of TPR motifs is conserved in NASP throughout the eukaryotic lineages. In this regard, NASP TPR analysis in kinetoplastids and parabasalids reveals that the TPR2 interruption is significantly smaller than the other family members and the acidic residues are either mostly N-terminal or dispersed throughout the protein, respectively. Phylogenies based on rRNA sequences have previously shown that excavata are one of the earliest diverging eukaryotes [46,49]. It seems reasonable therefore to assume that the interruption of TPR2 with acidic patches occurred later during eukaryotic evolution, after the diversification of excavata lineages e.g., parabasalids and kinetoplastids.

A phylogeny based on the functional units of a protein may provide insights into a protein’s functional diversification. The phylogeny shown in Figure 1 was reconstructed using conserved blocks of NASP TPR1-4 amino acid sequences, and indicates that NASP evolution largely parallels eukaryotic evolution. Existing genetic and biochemical evidence available for several well characterized NASP proteins including Hif1, Sim3, and sNASP reveal that in addition to being H3/H4 chaperones, these proteins exhibit some level of functional diversity. For example, Hif1 has been shown to physically interact in a stoichiometric manner with Hat1 and Hat2 in *S. cerevisiae* and is involved with acetylation of newly synthesized histone H4, whereas Sim3 which is a fission yeast NASP homolog appears to function specifically in the deposition of centromere-specific histone H3 variant CENP-A [14,20]. Different NASP proteins cluster together based on their taxonomic groups suggesting a lineage-specific functionalization pattern. Such clustering patterns along with varying branch lengths observed among different groups provide a good indication for the possible functional diversity. For example, our analysis suggests that *S. cerevisiae* and *T. phaffii* might be evolving faster in comparison to the other fungal lineages and may participate in lineage specific functions. A test of positive selection using branch site models may help reveal the identity of any residues that might have been positively selected in these lineages. Our phylogenetic (Figure 1) and sequence alignment (Additional file 3) analyses indicate that NASP is highly conserved among chordates consistent with a previous report which indicated that NASP antibody has high cross reactivity across different chordate species [34]. The choanoflagellate and amoebozoan NASP proteins are however highly divergent in comparison to the other crown group lineages and several key residues in the TPR motifs that are conserved in other lineages have changed. These amino acid substitutions might be functionally significant although this hypothesis awaits biochemical and structural analysis.

### Selective constraints operating on NASP

Estimation of amino acid *p*-distances suggests that TPR1 and TPR4 are evolving faster than TPR2 and TPR3 potentially accounting for the functional diversification observed among the NASP family members. This is further supported by our observation of more non-synonymous variations in TPR1/4 relative to TPR2/3 (Table 2). A deletion analysis of TPRs in a model system such as budding yeast [Fillingham et al. unpublished observations] that is amenable to a relatively straight forward biochemical analysis may provide further insights into specific TPR motif function. Comparison of synonymous and non-synonymous nucleotide substitutions indicated the presence of strong purifying selection that is operating at the protein level. This silent divergence indicates that NASP evolution has been subject to strong functional constraints maintaining essential structural features required for the protein’s proper functioning. Similar functional constraints have also been reported for other histone interacting proteins such as the nucleoplasmin (NPM) family of H2A/H2B histone chaperones [92]. Although the number of synonymous variations was always significantly higher than non-synonymous variations, by comparing the number of non-silent variations between the acidic and TPR regions, it is possible to assign significantly lower levels of non-silent variations in the latter. This indicates the presence of the strongest functional constraints on these regions operating in the form of purifying selection. Slightly higher levels of non-silent variations in the acidic regions could be due to the fact that selection for either aspartic acid or glutamic acid serves to maintain an overall negative charge.

Negatively charged residues found in NASP are important for the stability and proper functioning of the protein [16]. Selection has acted to maintain high frequency of these residues in the acidic region suggesting a deviation from neutrality. The neutral model of evolution predicts that the amino acid content of a protein is influenced by the nucleotide composition of its corresponding gene [93,94]. For example, the protein encoded by a gene with high GC bias will primarily be composed of amino acids that are encoded by GC-rich codons as a result of mutational bias. However, in contrast to the neutral model, the presence of strong selection at the protein level could alter the nucleotide composition bias. For NASP proteins, a deviation from neutrality is revealed when genomic GC content is compared with GC-rich and GC-poor residues. In contrast to the neutral model which predicts that GC-rich and GC-poor residues should have a positive and negative correlation with the genomic GC content, respectively, we observe no significant correlation between GC-rich/GC-poor residues and the genomic GC content. Comparison of the nucleotide frequencies at the first and third codon positions of lysine and alanine which comprise the most abundant residues in NASP provide insight into the role of natural selection. According to the neutral model, if the mutations are random and have equal probability of being fixed, then accordingly the four bases A, T, G, and C should occur with equal frequencies in the DNA [62,93]. Our results indicate that the frequency of G+A is significantly higher at the first codon position in comparison to the third codon position. This difference is particularly evident when frequencies are compared for the acidic domains. The nucleotide frequencies have therefore been influenced by strong selection for lysine, alanine, glutamic acid, and aspartic acid. Most studies at the genome level do not support selection as the major determinant of the amino acid composition [95-97]. However, it has been suggested that local scale deviations from neutrality are generally overlooked in studies that aim to focus on genome-wide patterns [98]. Accordingly, a few studies have shown that natural selection is more important than mutational biases in shaping the nucleotide/amino acid composition [92,98-100]. For example, in proteo-bacteria, high levels of alanine and lysine in the Tol A protein are maintained due to strong selective pressures [98]. Similarly, for the NPM family of histone chaperones, high levels of adenine at the second codon position occur due to a selection for amino acids lysine and/or glutamic acid and aspartic acid [92]. The present work, in addition to other studies, provides useful insights regarding the role of natural selection in determining the nucleotide/amino acid composition of a protein by demonstrating that elevated levels of G+A in NASP are maintained due to selection for alanine and/or glutamic acid/aspartic acid rather than the random mutational biases.

Several studies suggest that the unequal usage of synonymous codons exists because some codons are translated more efficiently and accurately and hence are the subject of selection (for review see [83]). We do not observe any significant codon bias among different NASP lineages (Table 3), although it has been shown that the sites that encode the most conserved and functionally important amino acids exhibit a greater bias in codon usage [60,61] due either to selection or mutational bias. Further work will be required to investigate whether or not this is also the case for NASP proteins among different lineages. The determination of the most frequently used synonymous codons that encode the conserved residues in NASP TPRs may provide some insight. GC bias has previously been shown to positively correlate with the extent of codon usage bias [85]. In the case of ray-finned fish-specific NASP paralogs, we observed that *NASP1* has higher GC content than *NASP2* and accordingly shows more biased trends in codon usage. These differences may represent the possible functional divergence between the two paralogs. Furthermore, GC content has been linked with elevated gene expression levels [84,86]. The comparison of gene expression of the two paralogs should provide further insight into their functional differentiation. The amino acid substitutions in the TPRs as well as the absence of the N-terminal region in NASP1 and the presence of the NNR domain in NASP2 provide good indications that functional diversity exists between the two ray-finned fish specific paralogs. A test of positive selection using the branch-site method as well as determination of interacting partners and sub-cellular localization for each paralogous protein will further elucidate the existence of sub- or neo-functionalization.

### Conserved pathway of H3/H4 transport

Transport of histones H3/H4 from the cytoplasm to the nucleus in yeast and human cells occurs in a stepwise fashion and is mediated by several proteins including heat-shock protein HSP90, NASP, Asf1 and Importinβ [10,12]. HSP90 and Asf1 have been shown to be highly conserved throughout the eukaryotes and most likely were present in the LECA [31,32,101]. A recent report established that the Karyopherin-β family of proteins is also highly conserved throughout the eukaryotic lineages and was well established prior to the LECA [33]. In this regard, the present work completes the evolutionary analyses of the major proteins involved in the transport of H3/H4 from the cytoplasm to the nucleus.

The physical interaction between HSP90-NASP, NASP-Asf1 and Asf1-Importinβ has been well described in humans and yeast [10,13]. Our recent report established that Nrp1-Asf1–Importinβ physical interactions are well-conserved in a more divergent eukaryote *T. thermophila*[28]. The evidence for the physical interaction between heat-shock protein and NASP is still restricted to Opisthokonta lineages. Our preliminary proteomic analysis of Nrp1 in *T. thermophila* indicates that this interaction is conserved [Nabeel-Shah and Fillingham, unpublished observations]. The predicted functional annotation of *T. thermophila* Nrp1 using the COFACTOR server suggests that TPR1 residues constitute the potential heat shock binding sites (Additional file 11). This prediction is of interest because human NASP TPR2 and TPR3/4 have been demonstrated to be critical for histones H1 and H3/H4-binding, respectively [16]. Nevertheless, the validity and generality of this prediction requires experimental verification.

In order to re-capitulate and extend previous work on Asf1 and Karyopherin-β family proteins we performed extensive database searches and identified putative Asf1 and Impβ orthologs throughout the major eukaryotes. This, combined with previous studies [31,33] suggests that these proteins were most likely present in the LECA. These studies indicate a conserved transport pathway of histones H3/H4 that most likely was present in the LECA and implicates HSP90, NASP, Asf1 and Importinβ proteins as the major components as their physical interactions have been described in divergent eukaryotic lineages including ciliates [28]. Other protein factors such as Hat1 complex, Codanin-1(human) and AIP1/AIP2 (ciliates) also play a role in histone H3/H4 transport [10,28,102]. However the evolutionary origin of their role in the pathway is unclear at present and the available biochemical evidence is limited to either one or a small subset of taxonomic groups.

## Conclusions

We have presented a detailed phylogenetic analysis of NASP family proteins. Several conclusions can be drawn from this study. First, NASP is widely distributed throughout the eukaryotes. It was most likely encoded in the genome of the last common ancestor of modern eukaryotes possibly representing an important innovation regarding the transport of H3/H4 from the cytoplasm to the nucleus. Second, natural selection has influenced the frequency of nucleotides encoding NASP in order to maintain high frequency of functionally important glutamic and aspartic acid amino acids. Third, TPRs1/4 are diverging more quickly relative to TPR2 and TPR4 possibly accounting for the functional diversity that has been reported among well characterized family members. Fourth, NASP paralogs found among ray finned fish potentially represent separate functional entities with NASP2 acquiring an NNR domain.

## Methods

### Sequence data retrieval and alignment

Amino acid sequences for well characterized NASP family members including *X. laevis* N1/N2 and human NASP were initially acquired from the NCBI protein database and were subsequently used as a query to search the non-redundant database with PSI-BLAST with default parameters. A phylogenomic approach was often used to increase the probability of identifying putative orthologs. For example, a putative NASP ortholog from the ciliate protozoan *T. thermophila* was used to detect further orthologs in other ciliate lineages. Sequences identified from each search were subsequently used as query for reciprocal recoveries. Protein sequences retrieved were analyzed at the Pfam database (http://pfam.sanger.ac.uk/) to examine the presence of SHNi-TPR (PF10516) [35]. However, SHNi-TPR for highly divergent lineages such as ciliates was delineated through sequence alignments. HMMprofile was prepared using hmmbuild and the UniProtKB database was scanned using hmmsearch from the HMMER package [37]. Nucleotide coding sequences were acquired from GenBank with the exception of sequences for fungal lineages which were extracted from the Broad Institute of genome database (http://www.broadinstitute.org). Nucleotide coding sequences for tNASP lineages and duplicated genes were obtained from sequenced genomes available at Ensemble v72 [103].

Protein multiple sequence alignments were built using MUSCLE with default parameters [104] and Jalview [105] was subsequently employed to inspect and edit the alignments. TPR 1–4 regions were predicted using well characterized human NASP TPR sequences as a template and were aligned against consensus TPR sequences. Secondary structure prediction and further domain analysis was carried out using the Protein homology/analogy recognition engine V 2.0 (PHYRE 2) [106]. The alignment of nucleotide sequences was built on the basis of their translated amino acid sequences using MUSCLE as implemented in software MEGAv5.2.1 [45].

For Asf1 and Importinβ, sequences for well characterized yeast and human proteins were retrieved from the NCBI database and were subsequently used as queries for BLAST searches with default settings to explore the non-redundant database. Retrieved sequences were aligned using MUSCLE and an HMMprofile was prepared as above. The UniProtKB database was scanned using HMMsearch limiting the search to eukaryotes only. For the karyopherin-β family the retrieved sequences were analysed for the presence of IBN_N domain (PF03810) and sequences lacking the domain or smaller than 500 amino acids were eliminated from the final data set, in accordance with [33]. Similarly Asf1 significant hits were checked for the presence of Pfam domain PF04729.

### Synteny analysis and GC content calculation

The Genomicus database v72.01 was queried using the human genome to investigate the genomic organisation of NASP family proteins [107]. DnaSP v5 was used to calculate GC content at the third codon positions [108].

### Molecular evolutionary and phylogenetic analyses

Protein phylogenetic analyses were accomplished under maximum likelihood (ML) and Bayesian frameworks using NASP amino acid sequences for 52 different eukaryotic lineages. MEGA v5.2.1 (MEGA) was used for inferring an ML tree whereas Bayesian analysis was carried out using Mr. Bayes v3.2.0 [109]. We carried out 6 different protein phylogenetic analyses using data sets consisting of: 1) combined amino acid sequences for all four predicted TPRs (excluding the acidic domain) in each protein; 2) entire protein sequences; 3) four analyses based on amino acid sequences for each of four individual TPRs. The analysis based on all four combined TPR sequences was conducted under rtREV+G+F as selected by MEGA and ProtTest v3.2 [44]. For the remaining five analyses the model of protein evolution that best fits the data was selected using MEGA. For entire protein sequences, rtREV+G+F was used whereas for individual TPRs 1–4 model WAG+G was selected for TPR1/2/4 and rtREV+G for TPR 3. For individual TPR2 phylogenetic analysis we also included the acidic amino acid patches that interrupt the TPR2 acidic domain. For this, alignment positions with more than 50% gaps were eliminated using trimAL before conducting the analysis. For each data set, a total of 1000 bootstrap replicates were run to obtain statistical support for the resulting ML tree. For Bayesian inferences, for each data set, two runs of 2 million generations were conducted with 0.25 burn-in frequency and resulting posterior probabilities were taken as indicators of phylogenetic reliability. FigTree v1.4.0 (http://tree.bio.ed.ac.uk/software/figtree/) and MEGA were used to visualize the resulting phylogenies.

Phylogenetic trees based on nucleotide coding sequences were reconstructed using ML, Bayesian and MP methods. A total of 1000 bootstrap replicates were run to obtain statistical support for the resulting MP and ML trees. ML analysis was carried out under the model GTR+G+I as selected by MEGA whereas MP trees were reconstructed using tree bisection and reconnection search method with search level 1 and with 10 replications for the random addition trees method. The ML phylogeny reconstruction to investigate the NASP paralogous relationships was conducted under the Tamura-Nei model with gamma distributions as selected by MEGA and statistical support was obtained based on 1000 replications. Bayesian analysis was conducted with two runs of 1 million generations (0.25 burn-in frequency) and resulting posterior probabilities were taken as indicators of phylogenetic reliability. For fish specific NASP paralogs, ancestral sequences corresponding to the pre-duplication node of the tree were inferred using MEGA under the empirical Bayesian method.

MEGA was used to analyse the nucleotide and amino acid compositions. To estimate the degree of amino acid and nucleotide divergence we used uncorrected differences (*p* distance) unless mentioned. This method was used because it gives better results than more complicated methods in particular for distantly related taxa due to its smaller variance. The modified Nei and Gojobori method [110] was used to compute the number of synonymous (*ps*) and non-synonymous (*p*_*N*_) nucleotide differences per site by providing transition/transversion ratios (R) in both cases. A complete deletion option was used to calculate the distances and the bootstrap method with 1000 replicates was employed for estimating the standard errors.

Several approaches were used to estimate the nature of selective forces operating on NASP evolution. A codon based Z-test of selection was carried out to study the presence and nature of selection. The alternative and null hypotheses were established as follows; H_**1**_: *p*_*N*_<*p*_*S*_ and H_**o**_:*p*_*N=*_*p*_*S*_. Probability to reject the null hypothesis and Z-test statistics were obtained. We tested deviation from neutrality by determining if selection has influenced certain amino acids. The GC content at fourfold degenerate sites was assumed to represent the genomic GC content providing that the latter has previously been shown to be a good approximation of the former [79,80]. In addition, GC content at four-fold degenerate sites was used as an approximation to the neutral expectation. Spearman rank correlation coefficient was used to compute the correlations and standard regression analysis was conducted for statistical significance. A comparison of nucleotide frequencies at the first codon position (always non-synonymous for the residues studied here) and at fourfold degenerate codon positions (always synonymous) was carried out in order to assess the influence of mutation and selection bias. Statistical significance of the results was also assessed by conducting student’s t test. In addition, estimation of the positive selection operating on individual codons was conducted using an ML-based method. We used the HyPhy program which is implemented in MEGA5. It involves the ML reconstructions of ancestral states under a Muse-Gaut model [111] of codon substitution. A nucleotide substitution model ‘GTR’ was also used as selected by MEGA and a user defined tree topology was provided. For detecting positively selected codons, the test statistic of dN –dS was used, where dS is the number of synonymous substitutions per site and dN is the number of nonsynonymous substitutions per site. Results were considered statistically significant for positive selection if the probability value was less than 0.05. Furthermore, the overall nucleotide variations (average number of synonymous and non-synonymous diversity per site) within TPRs 1–4 was estimated using a slide window approach with a window length of 50 bp and step size of 10 bp. The estimation of codon usage bias among NASP genes was conducted as the effective number of codons (ENC). Both analyses were carried out using the program DnaSP v5. In addition, relative synonymous codon usage (RSCU) [112] was estimated by MEGA using nucleotide coding sequences of the human *NASP* gene. To this end, an RSCU value greater than 1 indicates that a particular codon is used more frequently than expected whereas a value less than 1 indicates the reverse. Accordingly, an RSCU value of 1 indicates no codon bias [106].

### Structural bioinformatics

Tertiary structures for different NASP proteins were predicted using the I TASSER server (http://zhanglab.ccmb.med.umich.edu/I-TASSER/) [113] and subsequently were submitted to the web-PIPSA pipeline for estimating the electrostatic potentials. See Additional file 12 for details.

## Availability of supporting data

The data set supporting the results of this article is available in the TreeBase repository: http://purl.org/phylo/treebase/phylows/study/TB2:S15931[114]. Supporting data are also included as additional files.

## Competing interests

The authors declare that they have no competing interests.

## Authors’ contributions

SN-S retrieved the sequences, prepared sequence alignments, performed the molecular evolutionary analyses, conducted Bayesian phylogenetic analysis, and drafted the manuscript. KA performed ML and MP phylogenetic analyses, prepared the figures and conducted domain analysis using Pfam and PHYRE2. REP edited the manuscript and participated in design and co-ordination of the study and data analysis. JSF conceived the study, participated in its design and coordination, edited the manuscript and participated in data analysis. All authors have read and approved the final manuscript.

## Supplementary Material

Additional file 1 **Table S1.** Accession number of the sequences along with species name identified through PSI-BLAST. **Table S2.** Average number of amino acid and nucleotide variations among different regions of the NASP proteins discriminating various taxonomic groups. *p*AA, *p*NT, *p*S, and *p*N, represent the average number of amino acid, nucleotide, synonymous and non-synonymous nucleotide differences per site along with the Z-test of selection. SE indicates standard error based on 1000 bootstrap replicates. **Table S3:** Average number of amino acids among different TPRs discriminating various taxonomic groups. **Table S4:** Number of acidic residues (D + E) and theoretical iso-electric points distinguishing between the entire protein and acidic regions for the proteins used in this study **Table S5/6:** Average number of amino acid/nucleotide variations and Z-test of selection among different regions of the NASP paralogs. Click here for file

Additional file 2 **List of proteins identified in the study as putative NASP through HMMsearch.** Species names, accession numbers of UniProtKB and e-values are also provided. Click here for file

Additional file 3: Figure S1 A-E:SHNi-TPR sequence alignment of predicted TPR1 to TPR4 (A-D, respectively), across diverse eukaryotic lineages. The hydrophobic residues in each TPR that define the motif are denoted in black background. In accordance with Dunleavy et al. [35], the second residue in each TPR and 9^th^ residue for TPR3 (C) are highlighted with red indicating that residues at these sites are generally acidic or amidated. Gaps in the TPR2 (B) indicate that it is an interrupted form of TPR. TPR 4 (D) for certain lineages contains insertions in the linker regions that connect two helices in a TPR. These insertions are not shown, however their position is highlighted by underlining the residues and representing them in white against the blue background. Conserved residues are coloured according to the clustalX colour coding system [115]. E- Structural comparison based on TPR domain architect among different NASP proteins. Click here for file

Additional file 4: Figure S2 Phylogenetic tree of NASP proteins from different eukaryotic lineages reconstructed using entire protein sequences. Tree topology corresponds to bootstrap consensus ML tree reconstructed under the rtREV+G+F model. Branch lengths do not reflect genetic distance. Posterior probability values are indicated as underlined whereas bootstrap values based on 1000 replicates for the ML tree are indicated in light-face and are only reported when ≥ 50%. For Bayesian analysis the average standard deviation of split frequencies from two runs was 0.0072. Different taxonomic groups are indicated in the right margin. *Trypanosoma cruzi* and *Trypanosoma brucei* were used as out groups to root the tree. Click here for file

Additional file 5: Figure S3 A-D: Phylogenetic trees reconstructed using individual TPR motifs (TPR1-4 shown in A-D respectively) from different eukaryotic lineages. Tree topologies correspond to the bootstrap consensus ML trees and are shown in circular format. Branch lengths do not reflect genetic distance. Posterior probability values wherever possible are indicated as underlined whereas bootstrap values based on 1000 replicates for the ML tree are indicated in the light-face and are only reported when at least ≥ 50% (coloured clades). *Trypanosoma cruzi* and *Trypanosoma brucei* were used as out groups to root the trees in each case. Click here for file

Additional file 6: Figure S4 A. Phylogenetic relationship among different *NASP* orthologs. Trees were reconstructed using nucleotide coding sequences with ML and MP methods. The tree topology corresponds to bootstrap consensus ML estimations under the GTR+G+I model. Branch lengths do not reflect genetic distance. Confidence values for ML and MP trees are based on 1000 bootstrap replicates and are indicated (≥50%) in light-face and bold-face (underlined), respectively. *Trypanosoma cruzi* and *Trypanosoma brucei* were used as out groups to root the tree. B. Overall nucleotide diversity (Pi) among four TPRs corresponding to the lineages represented in Additional file 6: Figure S4A. A sliding window approach was used to calculate the overall synonymous/ non-synonymous differences per site. Click here for file

Additional file 7 **Figure S5.** GC content at the third codon position of NASP orthologs in various eukaryotic lineages. **Figure S6.** Relative Synonymous Codon Usage (RSCU) values for human NASP calculated using the nucleotide coding sequences. Different amino acids and their corresponding codons are grouped in different colors. **Figure S7.** Electrostatic distances calculated from the similarity indices for the electrostatic potentials of various NASP family proteins shown in a color-coded matrix heat map. The distance between similarity indices (S) of every pair of molecules (a and b) were calculated using $\;D_{a,b}=\sqrt{2-2S_{a,b}}$[116]. The color code and the density plot are also provided. Red and orange colors indicate similar potentials whereas more distant electrostatic potentials are represented with blue colors. Overall distances range from 0.04472 to 1.22229 (maximum range is from 0 to 2). The tree along the side of the image assembles the proteins into groups with similar electrostatic potentials (epogram). Despite the overall negative charge, it is apparent from the epogram that various NASP family members are interspersed suggesting that differences in electrostatic potentials are not the major selective force during NASP evolution. This signifies that maintaining a net negative charge through selection for acidic residues (D/E) has the functional importance rather than lineage specific set electrostatic potentials. Click here for file

Additional file 8: Figure S8 A. Phylogenetic relationship among different fish-specific NASP paralogs. Tree topology corresponds to the ML method under the JTT+G model as predicted by MEGA. Branch lengths do not reflect genetic distance. Alternatively, the same topology was also recovered by the neighbour joining method using p-distances. Confidence values for ML and NJ trees are based on 1000 bootstrap replicates and are indicated (≥50%) in light-face and boldface (underlined), respectively. The tree is rooted with *Halocynthia roretzi* and *Ciona intestinalis.* B-C: %GC3 content and ENC values are shown in chart diagrams for the fish-specific NASP1 and NASP2 neighbouring genes, respectively. The black line separates GC3 and ENC whereas blue and red colors represent different neighbouring genes as indicated in the chart legends. D-G**.** Predicted fish-specific NASP1/2 TPRs 1–4 sequence alignments are shown in figures B to E, respectively. Reconstructed ancestral sequence using the ML method is shown in black background whereas different amino acid substitutions in each TPR motif for two paralogous proteins are shown in blue. H-K. Predicted NASP1/2 TPRs 1–4 sequence alignments are shown in figures H to K, respectively, for various lineages. Click here for file

Additional file 9 **List of proteins identified in the study as putative Asf1 through a HMMsearch.** Species names, accession numbers of UniProtKB and e-values are provided. Click here for file

Additional file 10 **List of proteins identified in the study as putative Impβ through a HMMsearch.** Species names, accession numbers of UniProtKB and e-values are provided. Click here for file

Additional file 11 **A- Nrp1 predicted structure displayed in a surface representation complex with HSP90 peptide.** Functional annotation was carried out using the COFACTOR server (Predicted GO terms for molecular function and biological process are: GO: 0022892 and GO: 0006606, respectively). The interaction was predicted using the structure of the designed TPR module complex with HSP90 (PDB ID: 3KD7) as reported by Cortajarena et al. [117].The arrow indicates the position of the peptide. **B-** Detailed view of HSP90 peptide interaction with Nrp1 residues. HSP90 peptide is shown in grey whereas the predicted interacting residues are shown in stick representation. **C-** Predicted structural model of Nrp1 shown in ribbon representation. Predicted TPRs 1, 3 and 4 are highlighted in different colors whereas acidic residues interrupting TPR2 are indicated in CPK models. The position of a coiled-coiled domain (CCD) predicted by SMART is 150–189 and is not shown. The position of a second predicted CCD found towards the C-terminus is highlighted. **D-** Space-filling representation of Nrp1 structure indicating the degree of conservation as calculated by the program ConSurf [118] based on Nrp1 amino acid sequence aligned against 52 NASP proteins from different organisms using MUSCLE. The color key is provided. Click here for file

Additional file 12 Supplementary methods for structural prediction and calculation of electrostatic interaction properties. Click here for file
