# Supplementary material for: Molecular evolution of NASP and conserved histone H3/H4 transport pathway
Source: BMC Evol Biol. 2014 Jun 20;14:139. doi: 10.1186/1471-2148-14-139 (PMC4082323; doi:10.1186/1471-2148-14-139)
Supplement: Additional file 1 — Table S1. Accession number of the sequences along with species name identified through PSI-BLAST. Table S2. Average number of amino acid and nucleotide variations among different regions of the NASP proteins discriminating various taxonomic groups. pAA, pNT, pS, and pN, represent the average number of amino acid, nucleotide, synonymous and non-synonymous nucleotide differences per site along with the Z-test of selection. SE indicates standard error based on 1000 bootstrap replicates. Table S3: Average number of amino acids among different TPRs discriminating various taxonomic groups. Table S4: Number of acidic residues (D + E) and theoretical iso-electric points distinguishing between the entire protein and acidic regions for the proteins used in this study Table S5/6: Average number of amino acid/nucleotide variations and Z-test of selection among different regions of the NASP paralogs. [file 1471-2148-14-139-S1.pdf]

Supplementary Table1: Accession number of the sequences used in this study

**Species**	**Accession**	**Taxonomic Group**
Gallus gallus	CAG30998.1	Aves/Chordata
Mus musculus	NP_001074944.1	Mammalia/Chordata
Salmo salar	NP_001167378.1	Actinopterygii / Chordata
Xenopus laevis	AAI70018.1	Amphibia/Chordata
Xenopus laevis (V-2)	AAH77440.1	Amphibia/Chordata
Xenopus laevis (V-3)	CAA28419.1	Amphibia/Chordata
Xenopus tropicalis	NP_001164988.1	Amphibia/Chordata
Homo sapiens	NP_689511.2	Mammalia/Chordata
Bos taurus	XP_005204766.1	Mammalia/Chordata
Orcinus orca	XP_004285798.1	Mammalia/Chordata
Maylandia zebra	XP_004562165.1	Actinopterygii / Chordata
Taeniopygia guttata	XP_004174360.1	Aves/Chordata
Homo sapiens (tNASP)	NM_001195193.1	Mammalia/Chordata
Homo sapiens (tNASP-V2)	NM_002482.3	Mammalia/Chordata
Danio rerio	NP_956076.1	Actinopterygii / Chordata
Xiphophorus maculatus (NASP2)	ENSXMAG00000011059	Actinopterygii / Chordata
Xiphophorus maculatus (NASP1)	ENSXMAG00000018420	Actinopterygii / Chordata
Taeniopygia guttata	ENSTGUT00000008366	Aves/Chordata
Taeniopygia guttata (NASP2)	ENSTGUT00000017166	Aves/Chordata
Felis catus	XM_003990037.1	Mammalia/Chordata
Oryzias latipes (NASP2)	ENSORLG00000011095	Actinopterygii / Chordata
Felis catus (NASP2)	ENSFCAT00000023663	Mammalia/Chordata
Gorilla gorilla (tNASP)	ENSGGOT00000033308	Mammalia/Chordata
Gorilla gorilla (NASP2)	ENSGGOT00000028193	Mammalia/Chordata
Oryzias latipes (NASP1)	ENSORLG00000010548	Actinopterygii / Chordata
Gasterosteus aculeatus (NASP2)	ENSGACG00000006499	Actinopterygii / Chordata
Gasterosteus aculeatus (NASP1)	ENSGACG00000016167	Actinopterygii / Chordata
Oreochromis niloticus (NASP2)	ENSONIG00000008932	Actinopterygii / Chordata
Oreochromis niloticus (NASP1)	XP_003453360.1	Actinopterygii / Chordata
Takifugu rubripes (NASP2)	XP_003974435.1	Actinopterygii / Chordata
Takifugu rubripes (NASP1)	ENSTRUG00000012389	Actinopterygii / Chordata
Tetraodon nigroviridis (NASP2)	ENSTNIG00000018075	Actinopterygii / Chordata
Tetraodon nigroviridis (NASP1)	ENSTNIG00000006947	Actinopterygii / Chordata
Gadus morhua (Atlantic cod)(NASP2)	ENSGMOG00000008773	Actinopterygii / Chordata
Gadus morhua (Atlantic cod)(NASP1)	ENSGMOG00000011853	Actinopterygii / Chordata
Ictidomys tridecemlineatus (NASP1)	I3MSG5	Mammalia/Chordata
Ictidomys tridecemlineatus (NASP2)	I3NEY0	Mammalia/Chordata
Halocynthia roretzi	Q02508.1	Tunicates
Ciona intestinalis	XP_002120883.2	Tunicates
Tribolium castaneum	XP_970862.2	Arthropods
Megachile rotundata	XP_003708323.1	Arthropods
Danaus plexippus	EHJ78545.1	Arthropods
Apis florea	XP_003694981.1	Arthropods
Bombus terrestris	XP_003400722.1	Arthropods
Drosophila melanogaster	NP_649828.1	Arthropods

Drosophila sechellia	XP_002031992.1	Arthropods
Drosophila erecta	XP_001980917.1	Arthropods
Caenorhabditis elegans (NASP1)	NP_496380.1	Nematoda
Caenorhabditis elegans (NASP2)	NP_506298.1	Nematoda
Caenorhabditis remanei	XP_003117223.1	Nematoda
Caenorhabditis brenneri	EGT44801.1	Nematoda
Caenorhabditis briggsae (NASP1)	XP_002631180.1	Nematoda
Caenorhabditis briggsae (NASP2)	XP_002637622.1	Nematoda
Caenorhabditis brenneri (NASP2) **	EGT53840.1	Nematoda
Caenorhabditis remanei (NASP2) **	XP_003104429.1	Nematoda
Cryptococcus gattii	XP_003191095.1	Fungi
Trichosporon asahii var. asahii	EKD02263.1	Fungi
Tetrapisispora phaffii	XP_003687774.1	Fungi
Neurospora crassa	XP_961229.1	Fungi
Schizosaccharomyces pombe	NP_595313.1	Fungi
Coccidioides immitis	XP_001243327.1	Fungi
Saccharomyces cerevisiae	NP_013078.1	Fungi
Candida albicans	XP_712231.1	Fungi
Schizosaccharomyces japonicus	XP_002175640.1	Fungi
*Tremella fuciformis***	ADE10090.1	Fungi
*Candida glabrata***	XP_446527.1	Fungi
Arabidopsis thaliana	NP_568019.1	Plants
Capsella rubella	EOA15483.1	Plants
Theobroma cacao	EOX91255.1	Plants
Cicer arietinum	XP_004511989.1	Plants
Tetrahymena thermophila	XP_001030823.3	Ciliates
Ichthyophthirius multifiliis	XP_004027727.1	Ciliates
Paramecium tetraurelia	XP_001456110.1	Ciliates
Plasmodium yoelii	XP_729565.1	Apicomplexans
Neospora caninum	XP_003882797.1	Apicomplexans
Toxoplasma gondii	XP_002365332.1	Apicomplexans
*Babesia bovis***	XP_001609366.1	Apicomplexans
Albugo laibachii	CCA18837.1	Oomycete
Thalassiosira pseudonana	XP_002294415.1	Diatom
Choanoflagellida_Salpingoecidae	XP_004991759.1	*Choanoflagellates*
Dictyostelium fasciculatum	XP_004362161.1	Amoebozoa
*Dictyostelium discoideum***	XM_629896.1	Amoebozoa
*Acanthamoeba castellanii***	XP_004341330.1	Amoebozoa
*Acanthamoeba castellanii***	XM_004341282.1	Amoebozoa
*Nannochloropsis gaditana***	EKU20698.1	Alga
*Guillardia theta***	EKX34129.1	Cryptophyta
Trypanosoma cruzi	XP_810178.1	Kinetoplastids/Excavata
Trypanosoma brucei	CBH18038.1	Kinetoplastids/Excavata
*Trichomonas vaginalis***	A2DW29	Parabasalia/Trichomonadida /Excavata

* * Not included in the phylogeny reconstruction; V stands for variant

Supplementary Table2: Average number of amino acid and nucleotide variations among different regions of the NASP proteins discriminating various taxonomic groups

		p_{AA}(SE)	p_{NT}(SE)	p_S(SE)	p_N(SE)	R^a	Z-test[b]
Vertebrate	TPRs only	0.308±0.023	0.305±0.013	0.595±0.018	0.200±0.015	1.14	16.06***
	Acidic only	0.525±0.038	0.394±0.015	0.598±0.018	0.334±0.024	1.02#	7.216***
	Other Regions	0.439±0.023	0.388±0.011	1.670±0.078	0.403±0.032	0.92	14.75***
Tunicata	TPRs only	0.309±0.039	0.353±0.023	0.740±0.048	0.217±0.028	0.92	9.387***
	Acidic only	0.750±0.036	0.466±0.021	0.630±0.052	0.416±0.031	1.07#	3.381***
	Other Regions	0.507±0.042	0.480±0.023	0.796±0.056	0.385±0.033	0.69	5.938***
Arthropoda	TPRs only	0.511±0.022	0.426±0.013	0.60±0.018	0.361±0.019	1.19	7.541***
	Acidic only	0.648±0.021	0.524±0.011	0.639±0.017	0.490±0.016	0.89#	6.373***
	Other Regions	0.499±0.023	0.440±0.014	0.645±0.019	0.372±0.020	1.02	9.054***
Nematoda	TPRs only	0.350±0.029	0.337±0.017	0.630±0.032	0.230±0.023	1.13	9.583***
	Acidic only	0.728±0.043	0.531±0.029	0.757±0.054	0.473±0.037	0.65#	3.627***
	Other Regions	0.425±0.030	0.371±0.015	0.686±0.032	0.273±0.021	1.07	11.24***
Fungi	TPRs only	0.696±0.019	0.557±0.011	0.758±0.013	0.494±0.016	0.58	11.75***
	Acidic only	0.833±0.021	0.634±0.011	0.770 0.016	0.599±0.017	0.54#	6.551***
	Other Regions	0.768±0.024	0.605±0.013	0.779±0.018	0.556±0.021	0.58	7.472***
Plants	TPRs only	0.251±0.027	0.311±0.022	0.621±0.053	0.206±0.025	0.86	7.047***
	Acidic only	0.609±0.036	0.571±0.031	0.663±0.075	0.547±0.039	0.45	1.366*
	Other Regions	0.506±0.022	0.400±0.013	0.620±0.027	0.327±0.016	0.80	9.218***
Ciliates	TPRs only	0.593±0.030	0.451±0.017	0.661±0.039	0.395±0.024	0.69	5.116***
	Acidic only	0.862±0.033	0.444±0.026	0.546±0.058	0.422±0.038	0.53#	1.835**
	Other Regions	0.633±0.027	0.479±0.015	0.654±0.036	0.440±0.020	0.55	4.866***
Apicomplexa	TPRs only	0.685±0.022	0.519±0.013	0.686±0.023	0.467±0.019	0.71	7.066***
	Acidic only	0.779±0.039	0.529±0.032	0.729±0.051	0.461±0.042	0.69	3.358**
	Other Regions	0.646±0.019	0.528±0.012	0.687±0.022	0.476±0.017	0.72	6.887***
Euglenozoa	TPRs only	0.265±0.038	0.289±0.022	0.646±0.051	0.174±0.026	0.76	8.156***
	Other Regions	0.444±0.029	0.410±0.020	0.745±0.037	0.300±0.023	0.92	9.998***

p_{AA}, p_{NT}, p_S, and p_N represent average number of amino acid, nucleotide, synonymous and non-synonymous nucleotide differences per site along with Z-test of selection. SE indicates standard error based on 1000 bootstrap replicates.

[a] average transition/transversion ratio

Calculation performed using pair wise deletion

[b] H_1: $p_N < p_S$ and H_o: $p_N = p_S$

*** P< 0.001; ** P<0.05; * P<0.1

NOTE: Remaining region encompasses all sites excluding TPRs 1-4 and acidic interruption region of TPR2

Supplementary Table3: Average number of amino acid among different TPRs discriminating various taxonomic groups

	**TPR1(SE)**<br>*p*AA	**TPR2(SE)**<br>*p*AA	**TPR3(SE)**<br>*p*AA	**TPR4(SE)**<br>*p*AA
Vertebrate	0.313±0.043	0.392±0.03	0.273±0.049	0.428±0.048
Tunicata	0.559±0.086	0.400±0.047	0.206±0.070	0.235±0.069
Arthropoda	0.505±0.038	0.567±0.023	0.567±0.046	0.577±0.040
Nematoda	0.377±0.054	0.452±0.038	0.294±0.059	0.456±0.064
Fungi	0.761±0.027	0.741±0.022	0.646±0.041	0.663±0.043
Plants	0.284±0.055	0.404±0.034	0.206±0.048	0.284±0.062
Chromalveolata	0.824±0.021	0.669±0.034	0.694±0.037	0.805±0.022
Euglenozoa	0.412±0.084	0.180±0.054	0.294±0.076	0.235±0.073

*p*AA represents average number of amino acid variation and SE denotes standard error calculated based on 1000 bootstrap replicates

Supplementary Table 4: Number of acidic residues (Asp + Glu) and theoretical iso-electric points distinguishing between the entire protein and acidic regions for various NASP proteins

**Species**	**Whole protein (Asp + Glu)/ Theoretical pI**	**TPR2-Acidic region (Asp + Glu)/ Theoretical pI**
Tetrahymena thermophila	110/ 4.58	40/4.12
Saccharomyces cerevisiae	78/ 4.64	45/3.71
Coccidioides immitis	84/ 4.64	31/4.24
Schizosaccharomyces pombe	76/ 4.76	23/4.28
Neurospora crassa	105/ 4.58	41/4.49
Tetrapisispora phaffii	105/ 4.07	56/3.38
Trichosporon asahii	82/ 4.63	30/3.84
Cryptococcus gattii	79/ 4.40	28/3.63
Tribolium castaneum	79/ 4.42	38/4.09
Megachile rotundata	106/ 4.54	61/4.27
Danaus plexippus	86/ 4.46	42/3.85
Apis florea	113/ 4.50	68/4.22
Bombus terrestris	115/ 4.44	70/4.13
Drosophila melanogaster	102/ 4.30	48/3.89
Drosophila sechellia	103/ 4.30	49/3.88
Drosophila erecta	102/ 4.32	48/3.94
Danio rerio	164/ 4.40	111/4.14
Salmo salar	98/ 4.53	55/4.08
Oreochromis niloticus	139/ 4.37	88/4.12
Takifugu rubripes	105/ 4.37	52/4.19
Xenopus laevis	152/ 4.42	89/4.15
Xenopus tropicalis	155/ 4.38	93/4.11
Homo sapiens	99/ 4.35	35/3.95
Caenorhabditis elegans	95 /4.34	30/3.87
Caenorhabditis remanei	95 /4.31	30/3.93
Caenorhabditis brenneri	85 /4.30	31/3.99
Caenorhabditis briggsae	89 /4.38	23/3.98
Ichthyophthirius multifiliis	91 /4.81	24/4.26
Paramecium tetraurelia	86 /4.60	27/4.11
Halocynthia roretzi	119/ 4.67	54/4.40
Ciona intestinalis	117 /4.57	74/4.46
Mus musculus	98 /4.42	35/4.04
Gallus gallus	82 /4.79	36/3.94
Plasmodium yoelii	75 /4.59	19/4.41
Toxoplasma gondii	95 /4.59	17/4.50
Arabidopsis thaliana	96 /4.28	28/3.89
Capsella rubella	96 /4.47	28/4.04
Theobroma cacao	95 /4.37	33/4.12
Cicer arietinum	90 /4.46	27/4.22
Trypanosoma cruzi	100/ 4.48	-
Trypanosoma brucei	76 /4.60	-
Bos taurus	101/ 4.36	35/3.98
Orcinus orca	101 /4.36	35/3.98

Albugo laibachii	90 /4.84	18/4.40
Thalassiosira pseudonana	94 /4.51	23/3.66
Maylandia zebra	89 /4.26	37/3.75
Taeniopygia guttata	83 /4.86	36/3.92
Candida albicans	93 /4.41	36/3.84
Schizosaccharomyces japonicus	70 /4.69	22/4.38
Choanoflagellida Salpingoecidae	70 /4.85	32/3.98
Dictyostelium fasciculatum	87 /4.72	25/4.28
Neospora caninum	98/4.59	17/4.55

Supplementary Table5: Average number of amino acid and nucleotide variations among different regions of the NASP for various lineage specific paralogs

		p_{AA}(SE)	p_{NT}(SE)	p_S(SE)	p_N(SE)	R^a	Z-test[b]
Fish	TPRs only	0.232±0.023	0.253±0.011	0.525±0.019	0.152±0.016	1.03	13.68***
	Acidic only	0.383±0.039	0.329±0.019	0.577±0.032	0.251±0.030	0.93	8.326***
	Other Regions	0.344±0.030	0.309±0.014	0.555±0.022	0.225±0.020	0.93	12.05***
Nematoda	TPRs only	0.461±0.031	0.403±0.016	0.673±0.026	0.311±0.022	0.87	10.66***
	Acidic only	0.719±0.031	0.495±0.024	0.694±0.039	0.440±0.030	0.75	5.088***
	Other Regions	0.579±0.027	0.448±0.016	0.674±0.026	0.377±0.020	0.84	9.048***

p_{AA}, p_{NT}, p_S, and p_N, represent average number of amino acid, nucleotide, synonymous and non-synonymous nucleotide differences per site along with Z-test of selection. SE indicates standard error based on 1000 bootstrap replicates.

[a] average transition/transversion ratio

[b] H_1: $p_N < p_S$ and H_o: $p_N = p_S$

*** P< 0.001

Note: A pair wise comparison for each pair of sequences (NASP1/2) in these lineages was also carried out (not shown; See text for details).

Supplementary Table6: Average number of amino acid and nucleotide variations among different regions of the NASP for various lineage specific paralogs using pair wise comparisons

		p_{AA}(SE)	p_{NT}(SE)	p_S(SE)	p_N(SE)	R^a	Z-test[b]
I. tridecemlineatus Paralogs	TPRs only	0.187±0.035	0.117±0.016	0.169±0.035	0.096±0.018	1.68	N/A[Ŧ]
	Acidic only	0.178±0.045	0.132±0.022	0.207±0.060	0.111±0.029	0.93	1.488[*]
	Other Regions	0.211±0.027	0.105±0.011	0.095±0.020	0.107±0.014	2.13	N/A[Ŧ]
G. gorilla Paralogs	TPRs only	0.105±0.033	0.050±0.013	0.033±0.020	0.058±0.019	2.25	N/A[Ŧ]
	Acidic only[¥]	0.096±0.017	0.038±0.007	0.016±0.008	0.046±0.008	1.82	2.59[**]
	Other Regions	0.215±0.028	0.143±0.014	0.155±0.026	0.139±0.019	0.92	N/A[Ŧ]

p_{AA}, p_{NT}, p_S, and p_N represent average number of amino acid, nucleotide, synonymous and non-synonymous nucleotide differences per site along with Z-test of selection. SE indicates standard error based on 1000 bootstrap replicates.

[a] average transition/transversion ratio

[b] H_1: $p_N < p_S$ and H_o: $p_N = p_S$

[**] $P<0.05$; [*] $P<0.1$

Ŧ Null hypothesis of $p_N = p_S$ could not be rejected

¥ Test of Positive selection; H_1: $p_N > p_S$ and H_o: $p_N = p_S$

[**] $P<0.05$
