# Supplementary material for: Molecular evolution of NASP and conserved histone H3/H4 transport pathway
Source: BMC Evol Biol. 2014 Jun 20;14:139. doi: 10.1186/1471-2148-14-139 (PMC4082323; doi:10.1186/1471-2148-14-139)

Supplementary Figure S1
A

Homo sapiens	AKKL L GL GQK HL VMGDI PA AV NAFQEAAS L L GKK
Bos taurus	AKKL L GL GQK HL VMGDI PA AV NAFQEAAS L L GKK
Mus musculus	AKKL L GL GQK HL VMGDI PA AV NAFQEAAS L L GKK
Gallus gallus	SKKL L GL GQK HL VMGNI PA AV NAFQEAAS L L GKK
Xenopus laevis	AKRL MGA GQK HL VMKDV RS AV NLFQEASS L L AKQ
Xenopus tropicalis	AKKL MAT GQK HL VMKDV RS AV TSFQEASS L L AKK
Salmo salar	ANKL I GT GKR HL VMGDV VS AV NYLQEACG ML AKR
Danio rerio	AKKL I GT GSR HL VMGDV VS AV SVFQEACA ML AEK
Oreochromis niloticus	ANKL I GT GKK HL VMGK V VE AV STLQEACG ML AKK
Takifugu rubripes	AKKL V GA GNK YL VL GDV VS AV AVFQDACS ML AAK
Halocynthia roretzi	I QEC MGA GKK DL LC NDY SE AV NCFQEACT L L SGK
Ciona intestinalis	YAI NL AN GKK NL LC GDF PE AV ACFQATSA L QSER
Tribolium castaneum	PKEL F GQ GVR AY VL QDF DT AV KAFSKASE L L AAE
Danaus plexippus	PEEL L AA GRR HL AV RDY TS AA ETLATACE L L AKT
Bombus terrestris	AATAI SH GKR HL LV RDY TM AV TALAQACQ L L AEK
Megachile rotundata	PATAI SH GKR HL LV RDY TM AV TALAQACQ ML VEK
Apis florea	PATAI SH GKR HL LV RDY TM AV TALAQACQ L L AEK
Drosophila melanogaster	GKEL F SQ GSR NF LV KS Y DE AA DELSQVCQ L YEEV
Drosophila sechellia	GKEL F SQ GSR NF LV KS Y DE AA DELSQVCQ L YEEV
Drosophila erecta	GKEL F SQ GSR NF LV KS Y DE AA DELSQVCQ L YEEV
Schizosaccharomyces pombe	I EQL V TQ GNM AY AQ KNY EE AV DKYGQAL MQSESI
Trichosporon asahii	VAKL V SE GKK AI AL RQW EE GV GKYADAL DL QREL
Caenorhabditis elegans	LAEL L AA GRR AL KV NDI DK AS DSLSEATE L SSEI
Caenorhabditis remanei	LAEL L AN GRR EL RV NNN AE AS EI LSEAAE L SVEI
Caenorhabditis brenneri	LSEL L TA GRR AI RV NNI EE AS EI LSQATE L STEI
Caenorhabditis briggsae	AAEL L AA GRR AL KV SDY ET AT EALSEASE MI VEL
Tetrahymena thermophila	LESRL TE AVE CY KN KNF DQ AE NI FSDL MI YAVKF
Ichthyophthirius multifiliis	LEQQI EK VKE HI RK NEF ES AE EI FSDL MI DAVKY
Paramecium tetraurelia	AKEL F NE GKH NE AE VMMQG VMQQALAY YKDE LAV
Plasmodium yoelii	AQEL F DM GNL EF KE SK NYD VA AERFSMAVEK KVK
Neospora caninum	AETL L DE GKA LF RS RN WAK AA EYFSRAVERK VTE
Toxoplasma gondii	ADTL L GE GKT VF RS RN WAK AA EYFSRAVERK VAE
Saccharomyces cerevisiae	MERQV QI AKD LL AQ KK F LE AA KRCQQTL DS L PKD
Schizosaccharomyces japonicus	I DQL V VQ GNK AF SQ KHY EI AA EKYSDAL EV L EQK
Tetrapisispora phaffii	I KTL V VE GAK YT AN SDL AN AS KCYAEL LDLE SKT
Candida albicans	VAKL I SE GSR AY SS KDF DL AS EKYGEACE E YSKS
Coccidioides immitis	AELK RL AS AKE AI KDY NS AA DL YSRAVEI QAELN
Neurospora crassa	LADL S AH AAA QY AQ KNY DE AA ELYAQAAE MQAEM
Cryptococcus gattii	VEKL V AE GKK AI AL HE WEQ GV DRYATAL DR MRLL
Arabidopsis thaliana	AEEL T EK GSV FL KE ND F AE AV DCFSRAL EI RVAH
Capsella rubella	ADEL T EK GSV FL KE QD F GE AV DCFSRAL EI RVEH
Theobroma cacao	ADEL A EK GSK AF KE ND F AE AA DCFSRAL EI RVAH
Cicer arietinum	ADEL MD KEN KAM KD NDY GE AA DNYSRAL EI RVGH
Albugo laibachii	QDPRF QR GLC LL KE HDY EA AV TI FEDL LKTK VEL
Orcinus orca	AKKL L GL GQK HL VMGDI PA AV NAFQEAAS L L GKK
Thalassiosira pseudonana	SLPSH AL AKQ YL NT GQF NQ AL EVLEESL GE MHER
Maylandia zebra	AKKL I GT GKR HL VMGDV VS AV NVFQEACS ML AEK
Taeniopygia guttata	SKKL L GL GQK HL VMGNI PA AV NAFQEAAS L L GKK
Choanoflagellida Salpingoecidae	AEVF A AA KSL YH KD EV DQA GA I LSDLL ET MV EQF
Dictyostelium fasciculatum	MGKV K AA KAL LD DQ DY L SA CE LLGSSLSA L SPI Y
Trypanosoma cruzi	ANEAR AV GI A HY NN HR Y ED AL DI QYRVVR HF EKK
Trypanosoma brucei	AVAMR AV GL E LF NK HN Y EE AL DVQYRI VR YF ENK

B

Homo sapiens	GEAFFFYGKSLLELAR----LELAWDMLDLAKIIFKRQ
Bos taurus	GEAFFFYGKSLLELAR----LELAWDMLDLAKIIFKRQ
Mus musculus	GEAFFFYGKSLLELAR----LELAWDMLDLAKIIFKRQ
Orcinus orca	GEAFFFYGKSLLELAR----LELAWDMLDLAKIIFKRQ
Gallus gallus	AEAFFYYGKSLLELAR----LELPWDMLELAKVIYKRQ
Taeniopygia guttata	AEAFFYYGKSLLELAR----LELAWDMLELAKVIYKRQ
Xenopus laevis	AEAFYSYGMSLLELAR----LQLAWEMLDLCKIIFKRQ
Xenopus tropicalis	ADAFYSYGMSLLELAR----LQLAWEMLDLCKLIFKRQ
Salmo salar	GEAFFLCGKALLELAR----LQLAWEMLEVAKVIYKRK
Danio rerio	GEAFFFCGKALLELAR----LRLAWEMLEVAKVIYKRK
Maylandia zebra	GEAFFLCGKSLLELAR----LQLAWEMLEVAKVIYKRK
Oreochromis niloticus	GEAFFWYGKALLDLAR----LQLAWEMLEVAKVIYKRK
Takifugu rubripes	GEALFLCGKSLLELAR----LQLAWEMLEVAKVIYKRK
Halocynthia roretzi	CEAYYYYGVSLLELAR----MQLAWEMLELAKVLYKKH
Ciona intestinalis	AESFYFYGKALLELAR----MQLAWEMLELAKVLYQKK
Tribolium castaneum	GDVYLYYGKSLLELSR----LQVAWEVLELAKRIFTNR
Danaus plexippus	AEAYLWYGKSLLGLSR----LQLAWEMLDLSRNILQKR
Bombus terrestris	GEPYLLYGRALLGLAR----LQVAWEVLELAKLVLLKR
Megachile rotundata	GEPYLLYGRALLGLAR----LQVAWEVLELAKLVLLKR
Apis florea	GEPYLLYGRALLGLAR----LQVAWEVLELAKLVLLKR
Drosophila melanogaster	GQPLLLYAKALIAMAL----LQLAWEILEAAAQIFSRQ
Drosophila sechellia	GQPLLLYAKALIAMAL----LQLAWEILEAAAQIFSRQ
Drosophila erecta	GQPLLLYAKALIAMAL----LQLAWEILEAAAQIFSRQ
Caenorhabditis elegans	FDSLYYYGMATLELAK----MKLSWEILETARCIAAAK
Caenorhabditis remanei	FDACYYYGMASLEVAK----MKLAWELLETARCTAAAK
Caenorhabditis brenneri	FDAYYFYGMATLELAK----MRLAWELLETCRCIAIVK
Caenorhabditis briggsae	YEYYYYYGMATLELGK----MKLAWEVLENARCIAMAK
Tetrahymena thermophila	SKYFFFYGDMLLSKLE----LQLAWENLELSRKIYNQE
Ichthyophthirius multifiliis	SKYYYYYGDMLLTKQD----LQLAWENLELCKKIIYKN
Paramecium tetraurelia	AEYYYMYGTIIVLKLS----FHIAWENLEVARVILEKE
Plasmodium yoelii	REYYLCFADALLTKEE----EQLAFEMFEFSRKCYEML
Neospora caninum	STYYLWFGDALLTKEE----EELAFEMLEMAKRCLLKK
Toxoplasma gondii	STFYLWFGDALLTKEE----EELAFEMLEMAKQCLLKK
Albugo laibachii	APIYYEYGNALLSLVE----LELAWEMLEVARVIYTRH
Thalassiosira pseudonana	APLYYLYGTTLLYLVE----LQIAWENLETARSIMSKM
Schizosaccharomyces pombe	RNVLWLYGKSLFQIAI----DFNVAWEVLDLTRVMQSK
Trichosporon asahii	APLLLSYGKALYELAL----DFNAAWEVLDVARTIYTR
Saccharomyces cerevisiae	PELFTIFAQAVYNMEV----YENALDLLAQALMLLGRP
Schizosaccharomyces japonicus	RNVLWLYGRTLFEIAL----FGLAWEVLDLCRVLQTRA
Tetrapisispora phaffii	PDHVMLLASCLYQLGV----FQNTLELLQVARIIYMEN
Cryptococcus gattii	APLLLAYGKALYDLAS----YNAAWEVLDVARTIYQKI
Candida albicans	ADLLELYGKAVFQSGV----FEVAWELLDLARALFEEK
Coccidioides immitis	ADLLYSYGRCLYHVAV----FENAFETLDMARVLLSRQ
Neurospora crassa	AEILFLYGRALFKVGQ----LAVAFQVLDLAVLFEKKL
Arabidopsis thaliana	INAYYRYGLALLAKAQ----LDMAWKMLDIARVITDKQ
Capsella rubella	VNAYYRYGSALLEKAQ----LDMAWKMLDIARAITDKQ
Theobroma cacao	LKAYYLYGRALLYKAQ----LDLAWKMLDVARAIADKQ
Cicer arietinum	VHTYYKYGCALLYKAQ----LDLAWKMLDIARAIVEKQ
Choanoflagellida Salpingoecidae	ARVYQLYGLCLIASAR----VQVGWECLECARVIYSRL
Dictyostelium fasciculatum	APIYIKYGRALLFSYK----LEVAWDVLELARVIYEKN
Trypanosoma cruzi	GVYFLDYGLSQLRVLQ----LEACFINLDVARVCFQKQ
Trypanosoma brucei	GIYFLDYGLSQLRMIQ----LEACFTNLDVARVCFQKQ

C

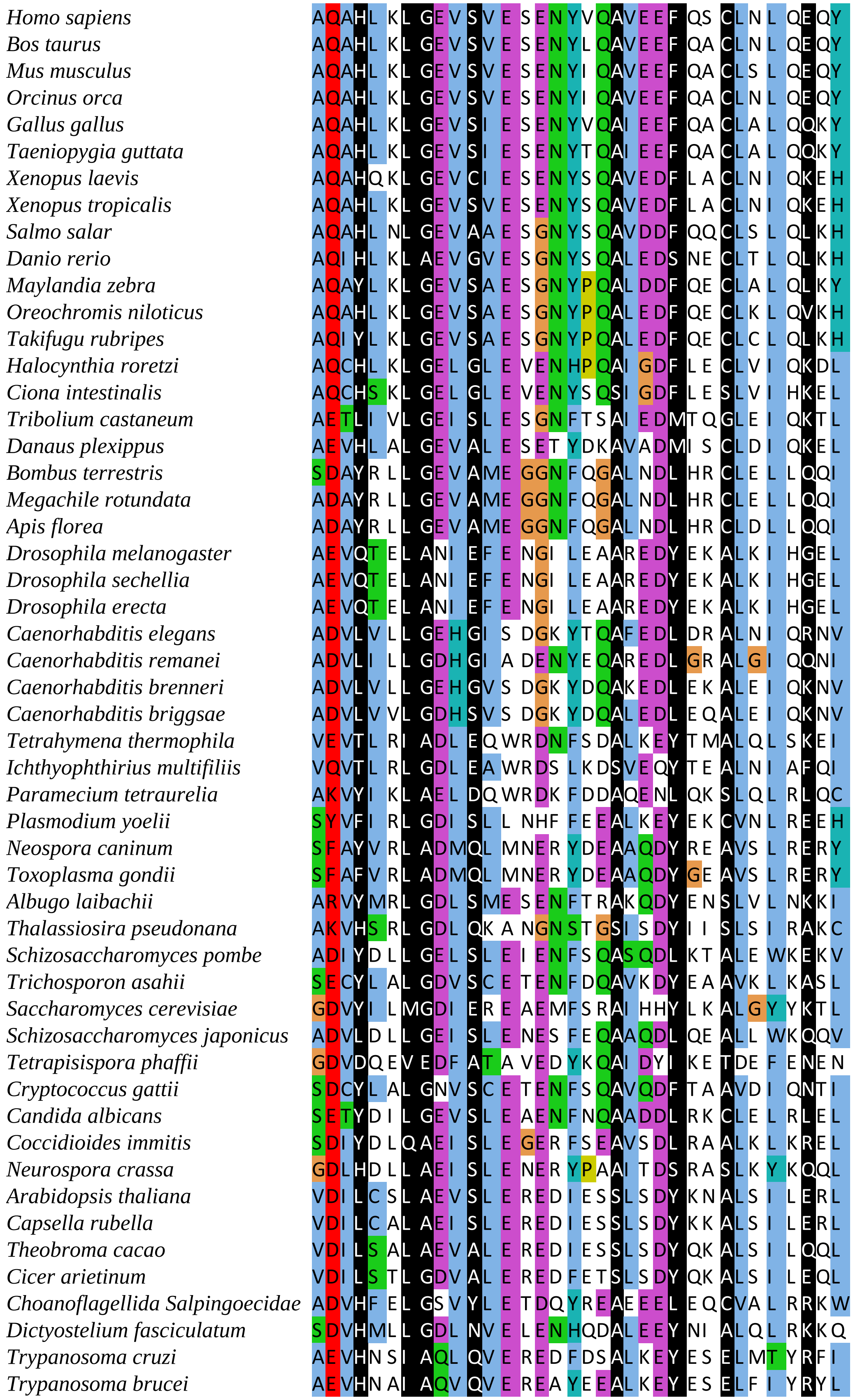
Homo sapiens
Bos taurus
Mus musculus
Orcinus orca
Gallus gallus
Taeniopygia guttata
Xenopus laevis
Xenopus tropicalis
Salmo salar
Danio rerio
Maylandia zebra
Oreochromis niloticus
Takifugu rubripes
Halocynthia roretzi
Ciona intestinalis
Tribolium castaneum
Danaus plexippus
Bombus terrestris
Megachile rotundata
Apis florea
Drosophila melanogaster
Drosophila sechellia
Drosophila erecta
Caenorhabditis elegans
Caenorhabditis remanei
Caenorhabditis brenneri
Caenorhabditis briggsae
Tetrahymena thermophila
Ichthyophthirius multifiliis
Paramecium tetraurelia
Plasmodium yoelii
Neospora caninum
Toxoplasma gondii
Albugo laibachii
Thalassiosira pseudonana
Schizosaccharomyces pombe
Trichosporon asahii
Saccharomyces cerevisiae
Schizosaccharomyces japonicus
Tetrapisispora phaffii
Cryptococcus gattii
Candida albicans
Coccidioides immitis
Neurospora crassa
Arabidopsis thaliana
Capsella rubella
Theobroma cacao
Cicer arietinum
Choanoflagellida Salpingoecidae
Dictyostelium fasciculatum
Trypanosoma cruzi
Trypanosoma brucei

D

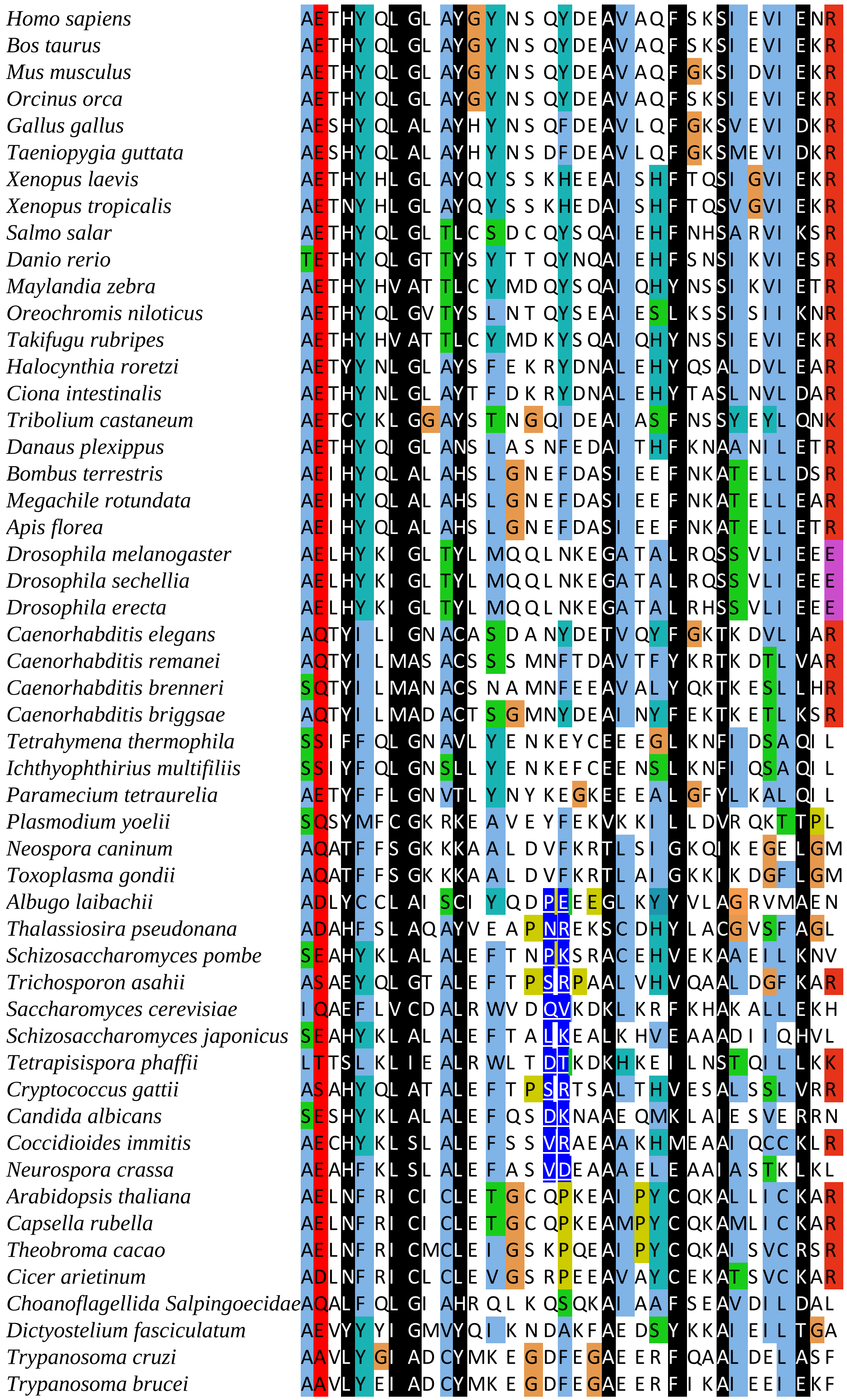
Homo sapiens
Bos taurus
Mus musculus
Orcinus orca
Gallus gallus
Taeniopygia guttata
Xenopus laevis
Xenopus tropicalis
Salmo salar
Danio rerio
Maylandia zebra
Oreochromis niloticus
Takifugu rubripes
Halocynthia roretzi
Ciona intestinalis
Tribolium castaneum
Danaus plexippus
Bombus terrestris
Megachile rotundata
Apis florea
Drosophila melanogaster
Drosophila sechellia
Drosophila erecta
Caenorhabditis elegans
Caenorhabditis remanei
Caenorhabditis brenneri
Caenorhabditis briggsae
Tetrahymena thermophila
Ichthyophthirius multifiliis
Paramecium tetraurelia
Plasmodium yoelii
Neospora caninum
Toxoplasma gondii
Albugo laibachii
Thalassiosira pseudonana
Schizosaccharomyces pombe
Trichosporon asahii
Saccharomyces cerevisiae
Schizosaccharomyces japonicus
Tetrapisispora phaffii
Cryptococcus gattii
Candida albicans
Coccidioides immitis
Neurospora crassa
Arabidopsis thaliana
Capsella rubella
Theobroma cacao
Cicer arietinum
Choanoflagellida Salpingoecidae
Dictyostelium fasciculatum
Trypanosoma cruzi
Trypanosoma brucei

Supplementary Figure S1-E

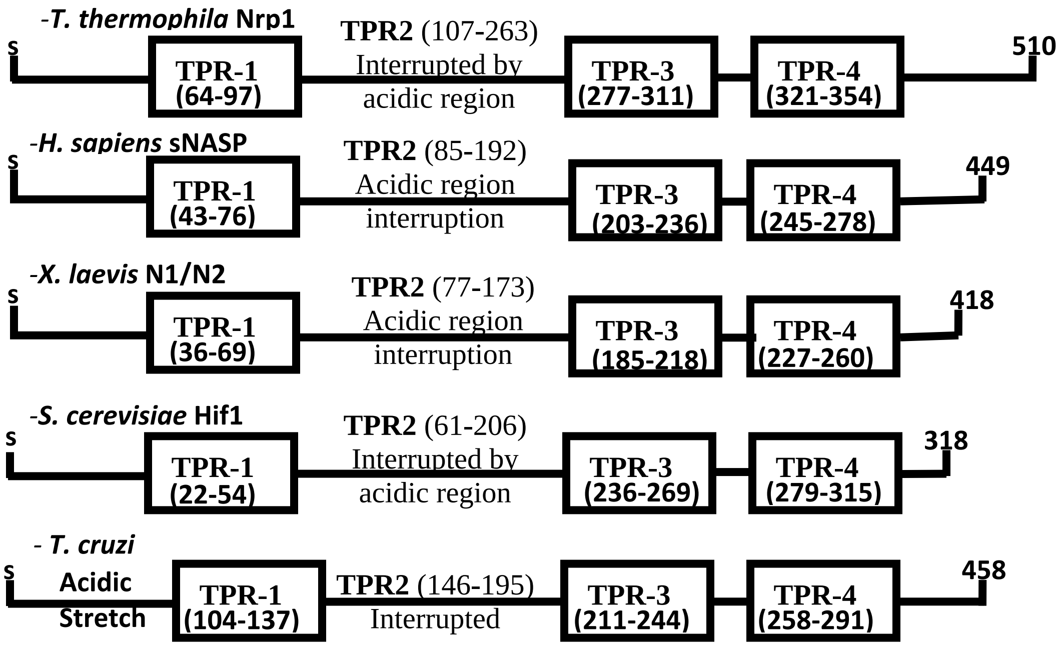

Supplement: Additional file 3: Figure S1 — A-E:SHNi-TPR sequence alignment of predicted TPR1 to TPR4 (A-D, respectively), across diverse eukaryotic lineages. The hydrophobic residues in each TPR that define the motif are denoted in black background. In accordance with Dunleavy et al. [35], the second residue in each TPR and 9th residue for TPR3 (C) are highlighted with red indicating that residues at these sites are generally acidic or amidated. Gaps in the TPR2 (B) indicate that it is an interrupted form of TPR. TPR 4 (D) for certain lineages contains insertions in the linker regions that connect two helices in a TPR. These insertions are not shown, however their position is highlighted by underlining the residues and representing them in white against the blue background. Conserved residues are coloured according to the clustalX colour coding system [115]. E- Structural comparison based on TPR domain architect among different NASP proteins. [file 1471-2148-14-139-S3.pdf]
