# Supplementary material for: Molecular evolution of NASP and conserved histone H3/H4 transport pathway
Source: BMC Evol Biol. 2014 Jun 20;14:139. doi: 10.1186/1471-2148-14-139 (PMC4082323; doi:10.1186/1471-2148-14-139)
Supplement: Additional file 4: Figure S2 — Phylogenetic tree of NASP proteins from different eukaryotic lineages reconstructed using entire protein sequences. Tree topology corresponds to bootstrap consensus ML tree reconstructed under the rtREV+G+F model. Branch lengths do not reflect genetic distance. Posterior probability values are indicated as underlined whereas bootstrap values based on 1000 replicates for the ML tree are indicated in light-face and are only reported when ≥ 50%. For Bayesian analysis the average standard deviation of split frequencies from two runs was 0.0072. Different taxonomic groups are indicated in the right margin. Trypanosoma cruzi and Trypanosoma brucei were used as out groups to root the tree. [file 1471-2148-14-139-S4.pdf]

Additional File 4: Figure S2: Phylogenetic tree of NASP proteins from different eukaryotic lineages reconstructed using entire protein sequences. Tree topology corresponds to bootstrap consensus ML tree reconstructed under the rtREV+G+F model. Branch lengths do not reflect genetic distance. Posterior probability values are indicated as underlined whereas bootstrap values based on 1000 replicates for the ML tree are indicated in light-face and are only reported when at least ≥ 50%. For Bayesian analysis the average standard deviation of split frequencies from two runs was 0.0072. Different taxonomic groups are indicated in the right margin. *Trypanosoma cruzi* and *Trypanosoma brucei* were used as out groups to root the tree.

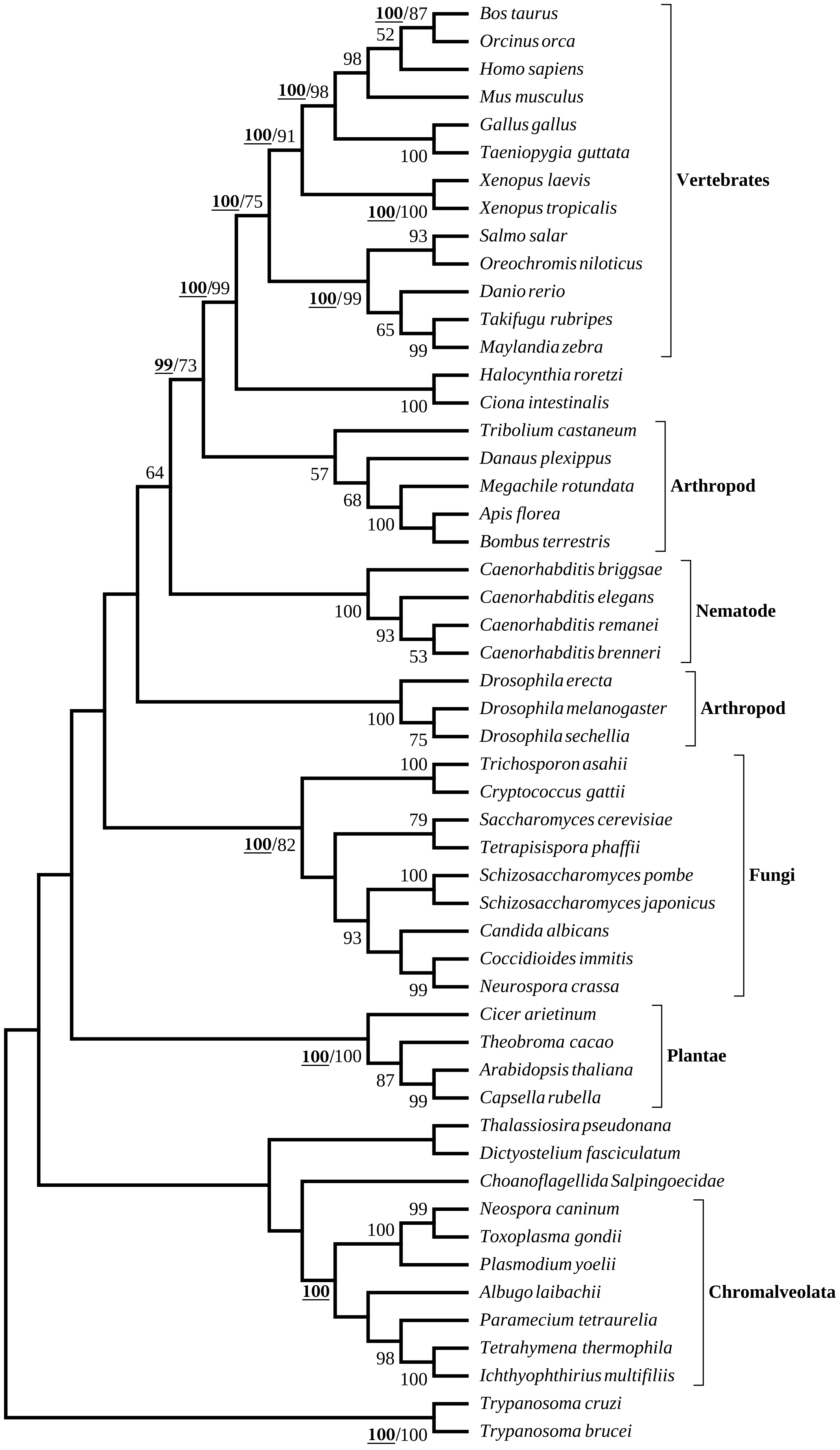

Bos taurus
Orcinus orca
Homo sapiens
Mus musculus
Gallus gallus
Taeniopygia guttata
Xenopus laevis
Xenopus tropicalis
Salmo salar
Oreochromis niloticus
Danio rerio
Takifugu rubripes
Maylandia zebra
Halocynthia roretzi
Ciona intestinalis
Tribolium castaneum
Danaus plexippus
Megachile rotundata
Apis florea
Bombus terrestris
Caenorhabditis briggsae
Caenorhabditis elegans
Caenorhabditis remanei
Caenorhabditis brenneri
Drosophila erecta
Drosophila melanogaster
Drosophila sechellia
Trichosporon asahii
Cryptococcus gattii
Saccharomyces cerevisiae
Tetrapisispora phaffii
Schizosaccharomyces pombe
Schizosaccharomyces japonicus
Candida albicans
Coccidioides immitis
Neurospora crassa
Cicer arietinum
Theobroma cacao
Arabidopsis thaliana
Capsella rubella
Thalassiosira pseudonana
Dictyostelium fasciculatum
Choanoflagellida Salpingoecidae
Neospora caninum
Toxoplasma gondii
Plasmodium yoelii
Albugo laibachii
Paramecium tetraurelia
Tetrahymena thermophila
Ichthyophthirius multifiliis
Trypanosoma cruzi
Trypanosoma brucei
Vertebrates
Arthropod
Nematode
Arthropod
Fungi
Plantae
Chromalveolata
100/87
52
98
100/98
100/91
100
100/75
100/100
93
100/99
100/99
65
99
99/73
100
64
57
68
100
100
93
53
100
75
100
79
100/82
100
93
99
100/100
87
99
99
100
100
98
100
100/100
