# Supplementary material for: Molecular evolution of NASP and conserved histone H3/H4 transport pathway
Source: BMC Evol Biol. 2014 Jun 20;14:139. doi: 10.1186/1471-2148-14-139 (PMC4082323; doi:10.1186/1471-2148-14-139)

Additional File 5: Figure S3- A-D: Phylogenetic trees reconstructed using individual TPR motifs (TPR1-4 shown in A-D respectively) from different eukaryotic lineages. Tree topologies correspond to the bootstrap consensus ML trees and are shown in circular format. Branch lengths do not reflect genetic distance. Posterior probability values wherever possible are indicated as underlined whereas bootstrap values based on 1000 replicates for the ML tree are indicated in the light-face and are only reported when at least ≥ 50% (coloured clades). *Trypanosoma cruzi* and *Trypanosoma brucei* were used as out groups to root the trees in each case.

Supplementary Figure S3A

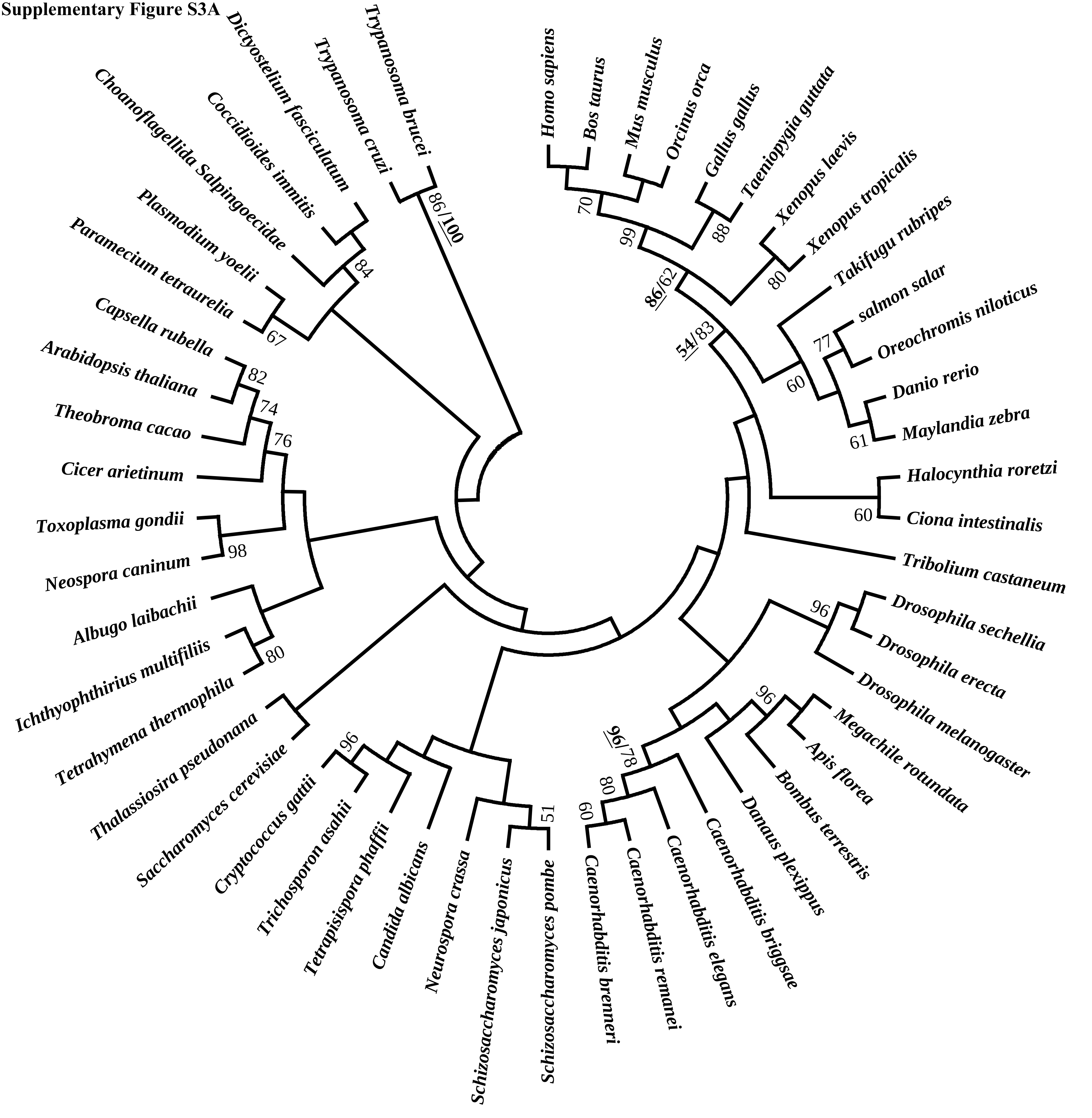

Supplementary Figure S3B

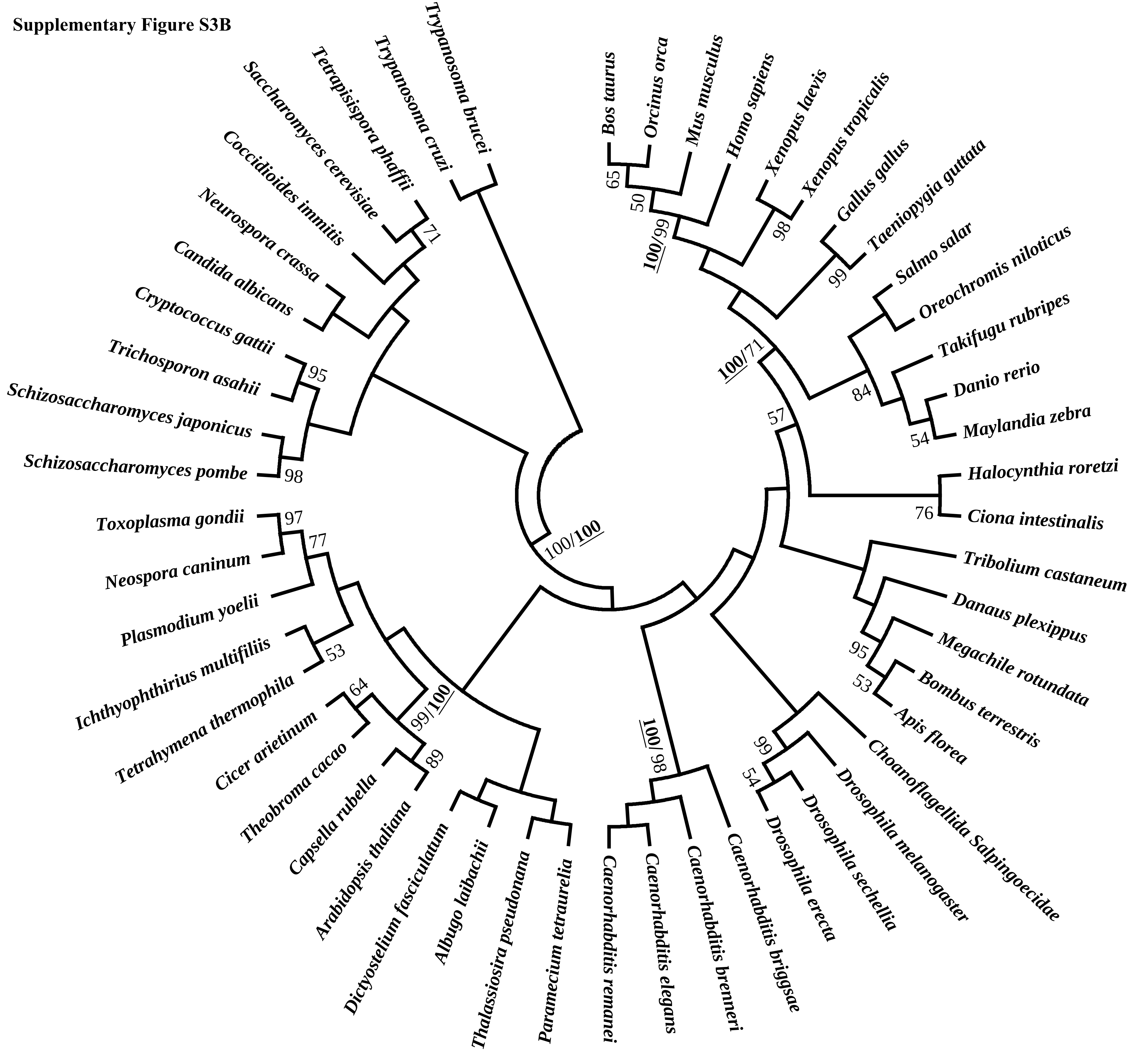

Supplementary Figure S3C

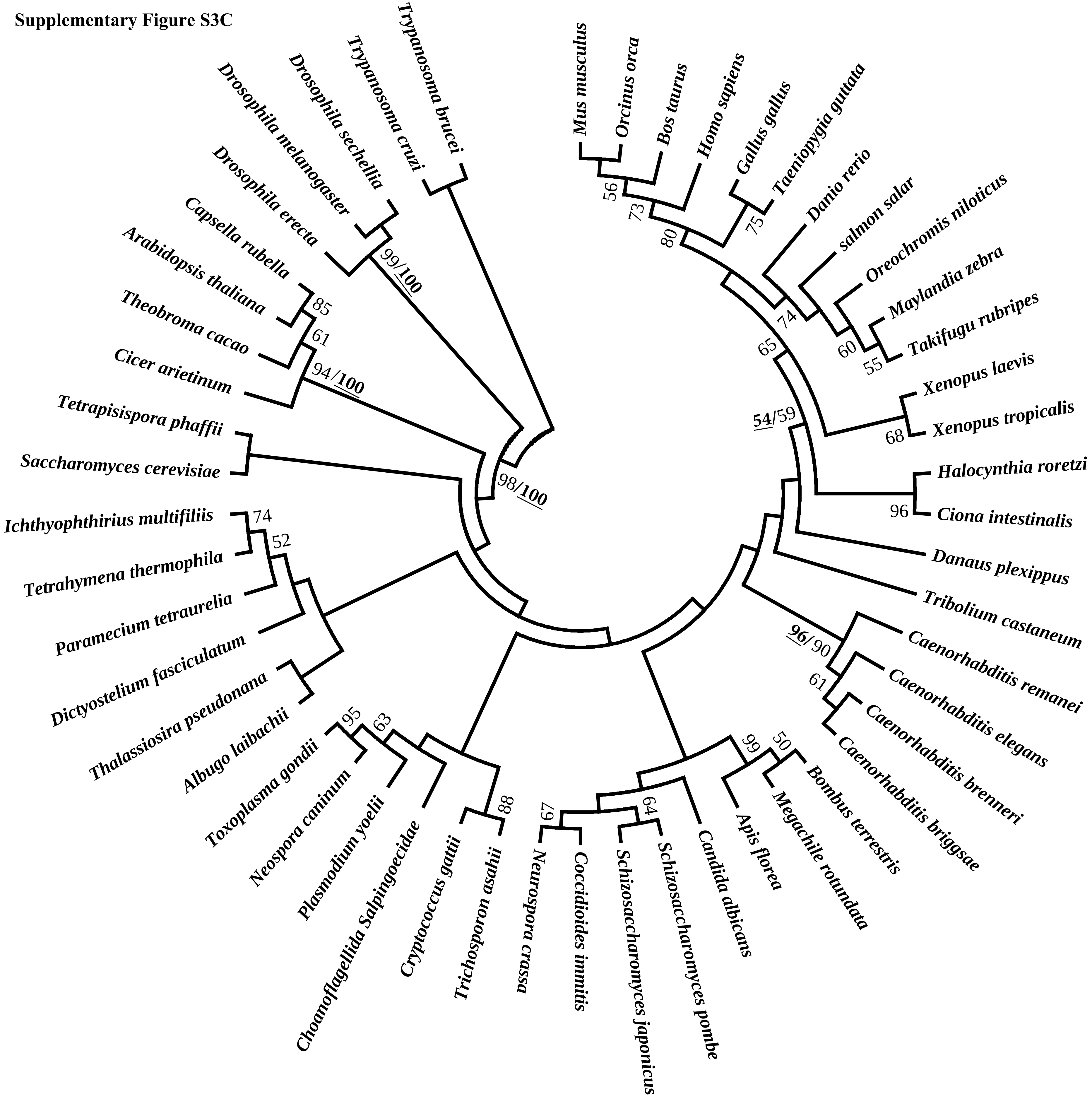

Supplementary Figure S3D

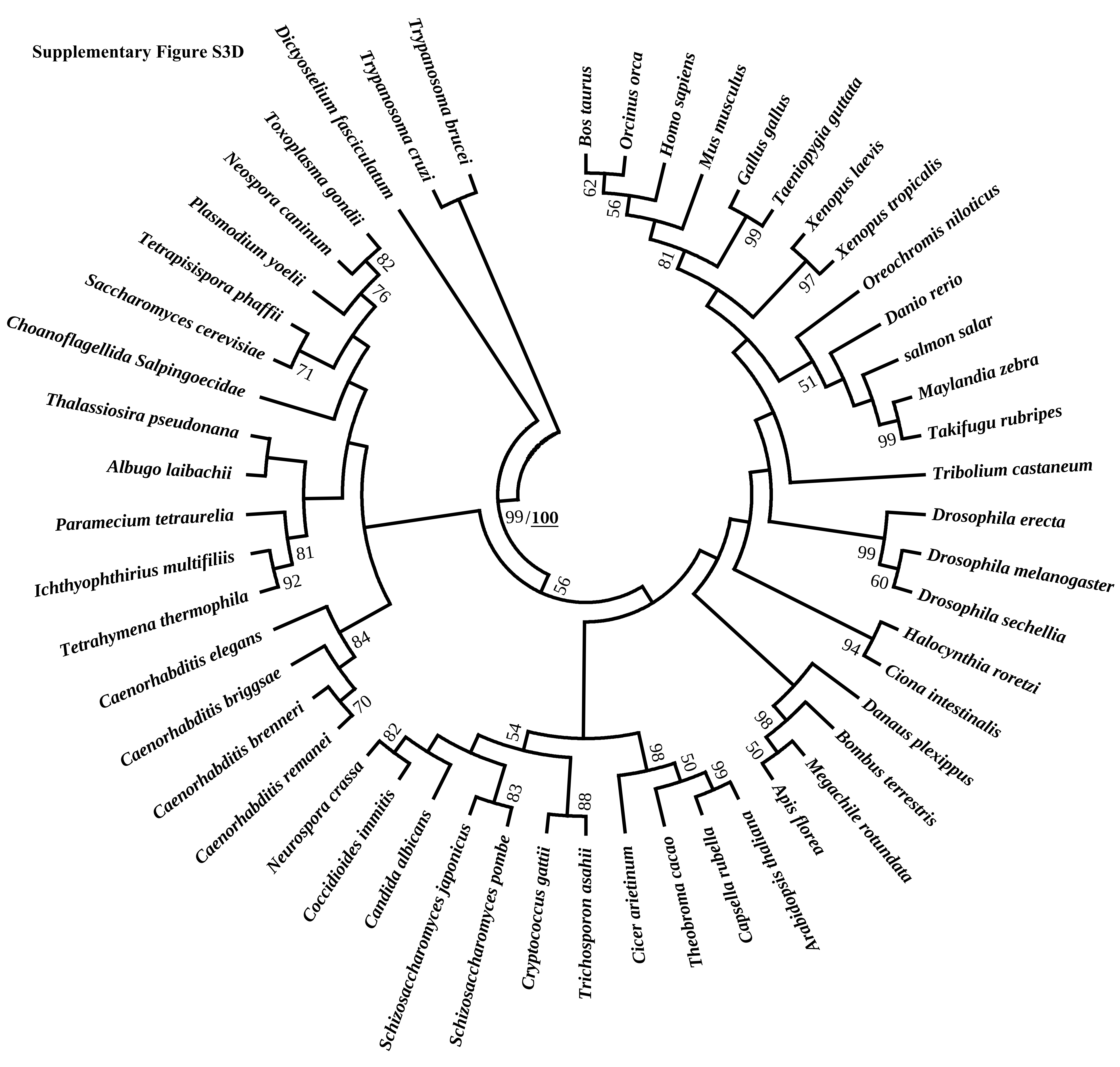

Supplement: Additional file 5: Figure S3 — A-D: Phylogenetic trees reconstructed using individual TPR motifs (TPR1-4 shown in A-D respectively) from different eukaryotic lineages. Tree topologies correspond to the bootstrap consensus ML trees and are shown in circular format. Branch lengths do not reflect genetic distance. Posterior probability values wherever possible are indicated as underlined whereas bootstrap values based on 1000 replicates for the ML tree are indicated in the light-face and are only reported when at least ≥ 50% (coloured clades). Trypanosoma cruzi and Trypanosoma brucei were used as out groups to root the trees in each case. [file 1471-2148-14-139-S5.pdf]
