# Supplementary material for: Molecular evolution of NASP and conserved histone H3/H4 transport pathway
Source: BMC Evol Biol. 2014 Jun 20;14:139. doi: 10.1186/1471-2148-14-139 (PMC4082323; doi:10.1186/1471-2148-14-139)
Supplement: Additional file 6: Figure S4 — A. Phylogenetic relationship among different NASP orthologs. Trees were reconstructed using nucleotide coding sequences with ML and MP methods. The tree topology corresponds to bootstrap consensus ML estimations under the GTR+G+I model. Branch lengths do not reflect genetic distance. Confidence values for ML and MP trees are based on 1000 bootstrap replicates and are indicated (≥50%) in light-face and bold-face (underlined), respectively. Trypanosoma cruzi and Trypanosoma brucei were used as out groups to root the tree. B. Overall nucleotide diversity (Pi) among four TPRs corresponding to the lineages represented in Additional file 6: Figure S4A. A sliding window approach was used to calculate the overall synonymous/ non-synonymous differences per site. [file 1471-2148-14-139-S6.pdf]

Additional File 6: Figure S4. A: Phylogenetic relationship among different *NASP* orthologs. Trees were reconstructed using nucleotide coding sequences with ML and MP methods. The tree topology corresponds to bootstrap consensus ML estimations under the GTR+G+I model. Branch lengths do not reflect genetic distance. Confidence values for ML and MP trees are based on 1000 bootstrap replicates and are indicated (≥ 50%) in light-face and bold-face (underlined), respectively. *Trypanosoma cruzi* and *Trypanosoma brucei* were used as out groups to root the tree. **B:** Overall nucleotide diversity among four TPRs corresponding to the lineages represented in figure S4A. A sliding window approach was used to calculate the overall synonymous/ non-synonymous differences per site.

Figure S4A

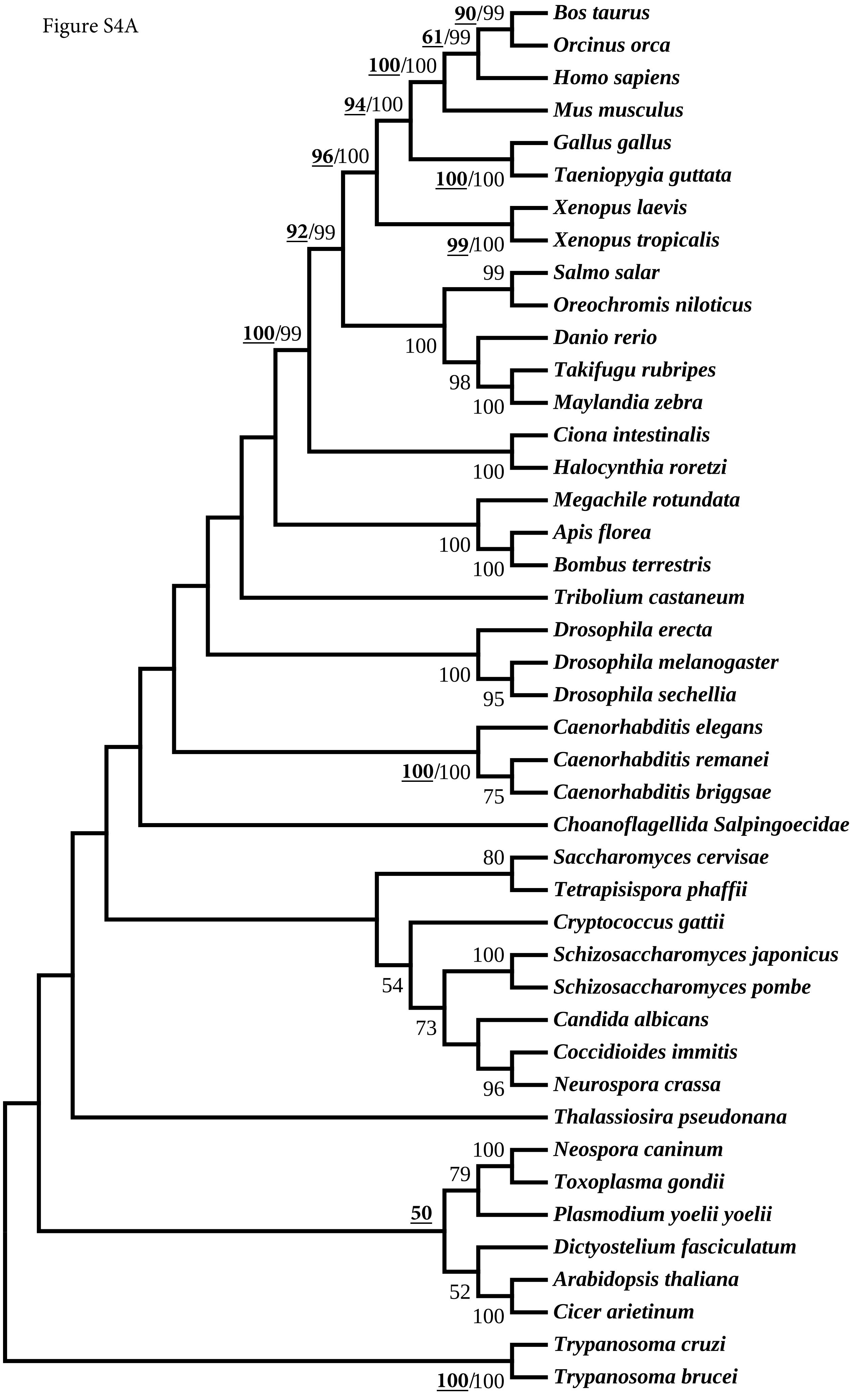

90/99
61/99
100/100
94/100
96/100
92/99
100/99
Bos taurus
Orcinus orca
Homo sapiens
Mus musculus
Gallus gallus
Taeniopygia guttata
100/100
Xenopus laevis
Xenopus tropicalis
99/100
99
Salmo salar
Oreochromis niloticus
100
Danio rerio
Takifugu rubripes
98
Maylandia zebra
100
Ciona intestinalis
Halocynthia roretzi
100
Megachile rotundata
Apis florea
100
Bombus terrestris
100
Tribolium castaneum
Drosophila erecta
Drosophila melanogaster
100
Drosophila sechellia
95
Caenorhabditis elegans
Caenorhabditis remanei
100/100
Caenorhabditis briggsae
75
Choanoflagellida Salpingoecidae
80
Saccharomyces cervisae
Tetrapisispora phaffii
Cryptococcus gattii
100
Schizosaccharomyces japonicus
Schizosaccharomyces pombe
54
Candida albicans
73
Coccidioides immitis
Neurospora crassa
96
Thalassiosira pseudonana
100
Neospora caninum
79
Toxoplasma gondii
50
Plasmodium yoelii yoelii
Dictyostelium fasciculatum
Arabidopsis thaliana
52
Cicer arietinum
100
Trypanosoma cruzi
Trypanosoma brucei
100/100

Figure S4B

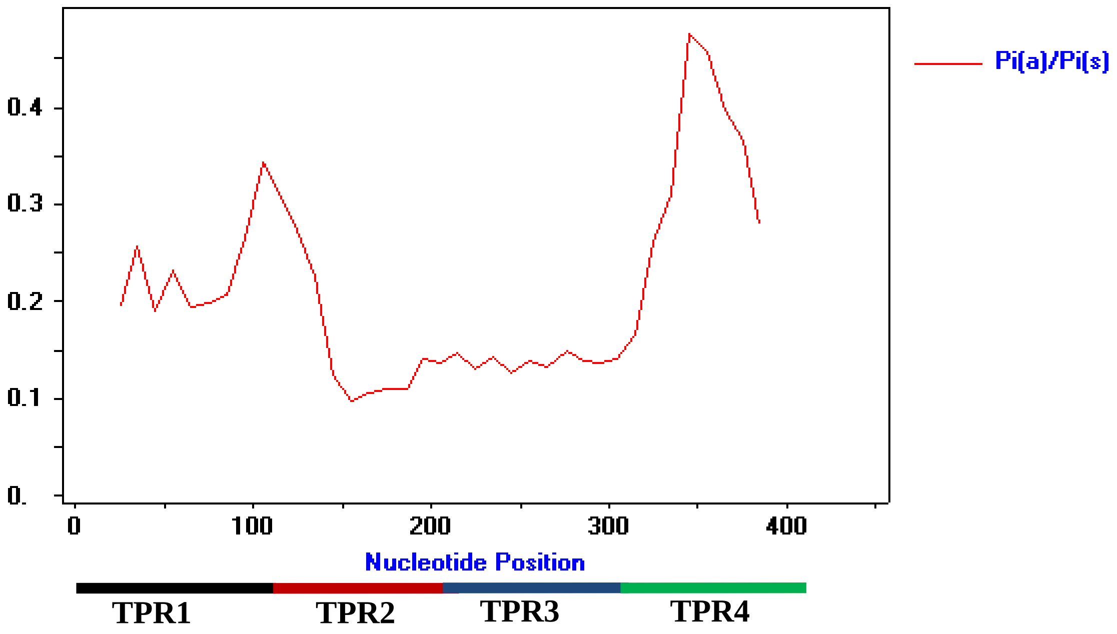
Pi(a)/Pi(s)
0.4
0.3
0.2
0.1
0.
0
100
200
300
400
Nucleotide Position
TPR1
TPR2
TPR3
TPR4
