# Supplementary figures and images for: Molecular evolution of NASP and conserved histone H3/H4 transport pathway
Source: BMC Evol Biol. 2014 Jun 20;14:139. doi: 10.1186/1471-2148-14-139 (PMC4082323; doi:10.1186/1471-2148-14-139)

Supplementary Figure S5

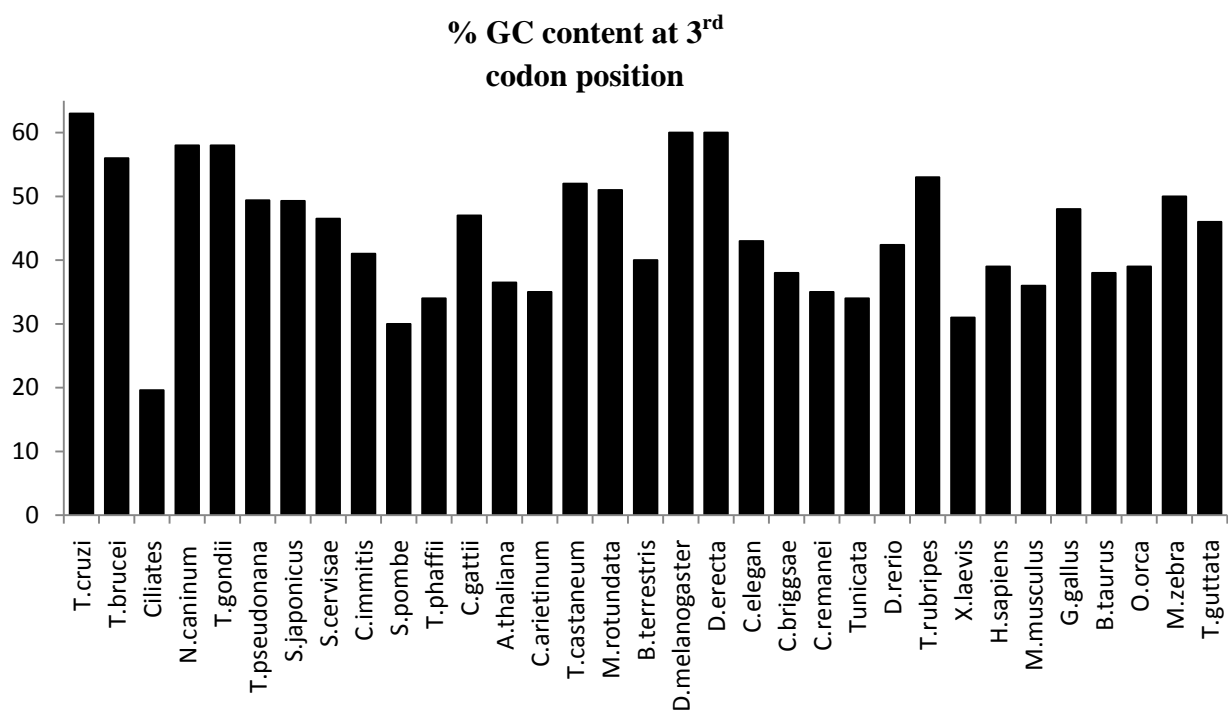

Supplementary Figure S6

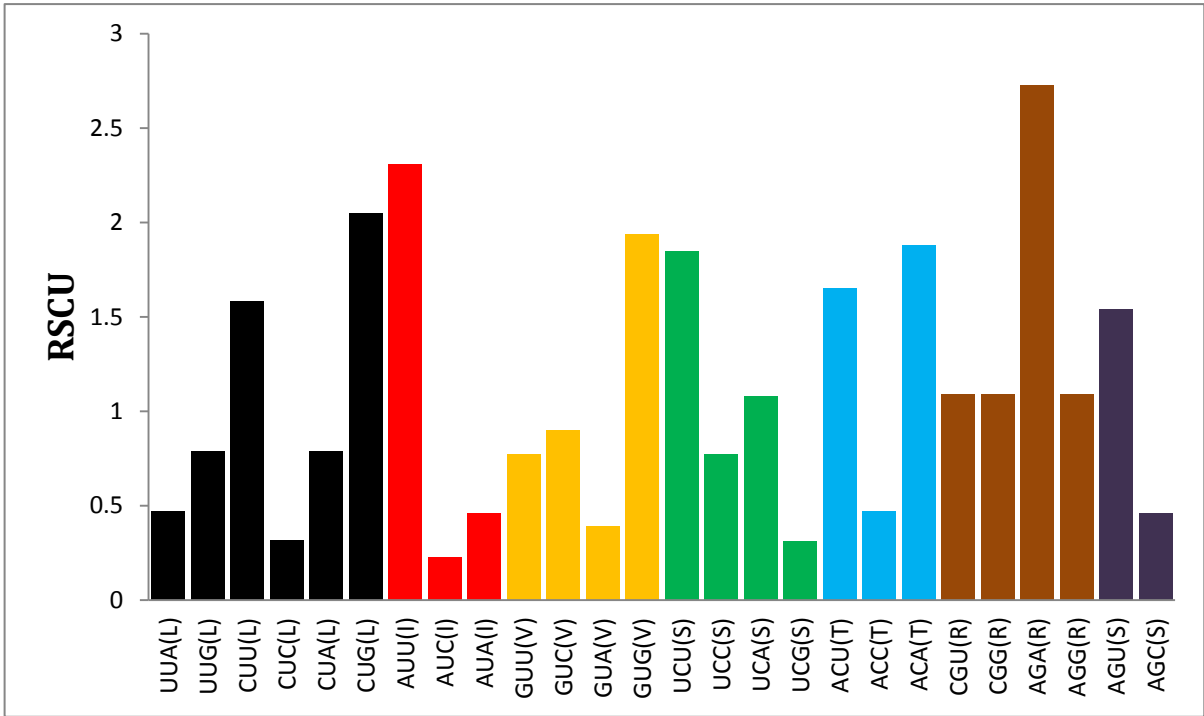

Supplementary Figure S7

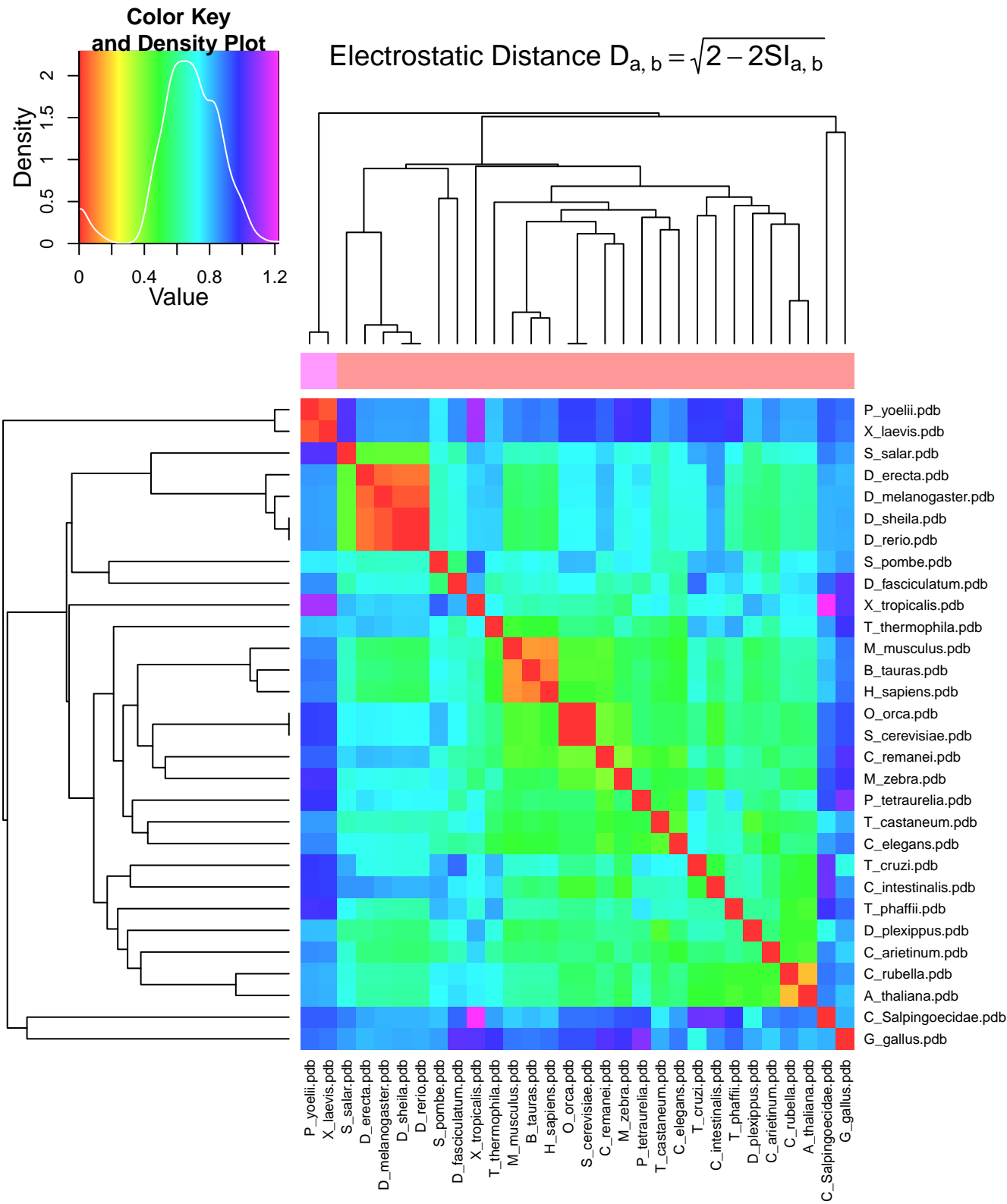

Supplement: Additional file 7 — Figure S5. GC content at the third codon position of NASP orthologs in various eukaryotic lineages. Figure S6. Relative Synonymous Codon Usage (RSCU) values for human NASP calculated using the nucleotide coding sequences. Different amino acids and their corresponding codons are grouped in different colors. Figure S7. Electrostatic distances calculated from the similarity indices for the electrostatic potentials of various NASP family proteins shown in a color-coded matrix heat map. The distance between similarity indices (S) of every pair of molecules (a and b) were calculated using Da,b=2-2Sa,b[116]. The color code and the density plot are also provided. Red and orange colors indicate similar potentials whereas more distant electrostatic potentials are represented with blue colors. Overall distances range from 0.04472 to 1.22229 (maximum range is from 0 to 2). The tree along the side of the image assembles the proteins into groups with similar electrostatic potentials (epogram). Despite the overall negative charge, it is apparent from the epogram that various NASP family members are interspersed suggesting that differences in electrostatic potentials are not the major selective force during NASP evolution. This signifies that maintaining a net negative charge through selection for acidic residues (D/E) has the functional importance rather than lineage specific set electrostatic potentials. [file 1471-2148-14-139-S7.pdf]

A

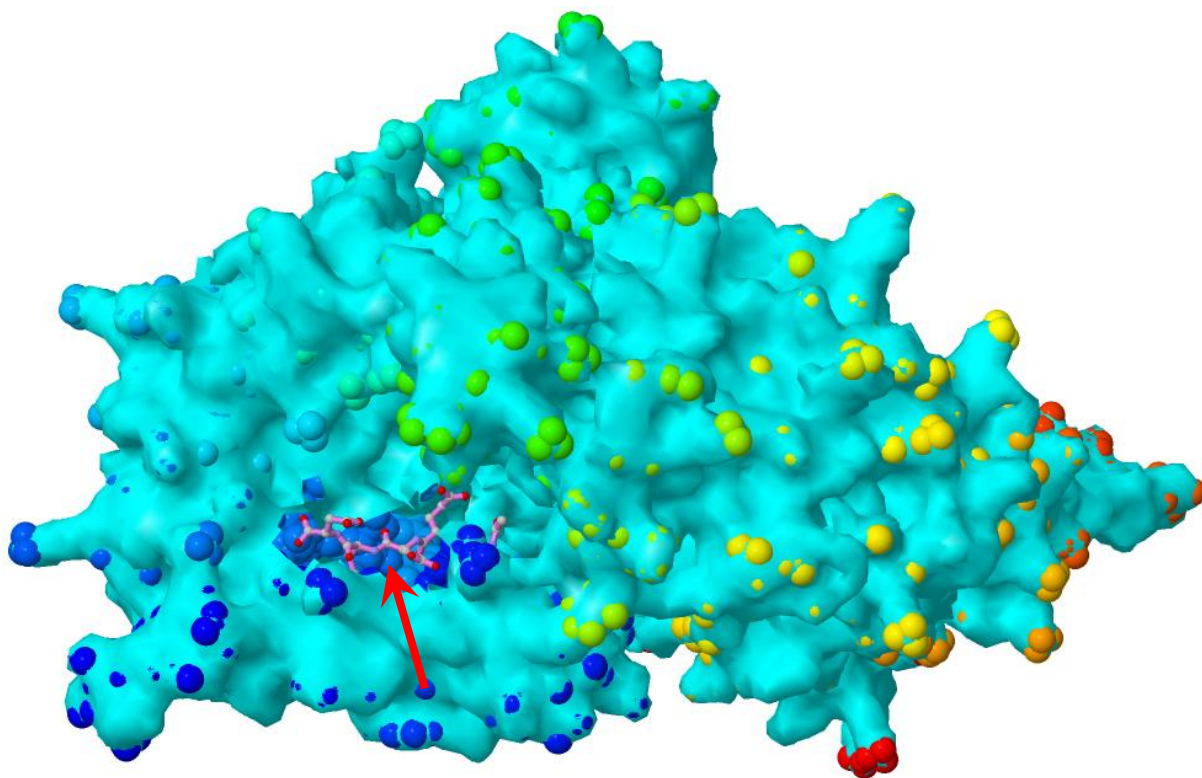

B

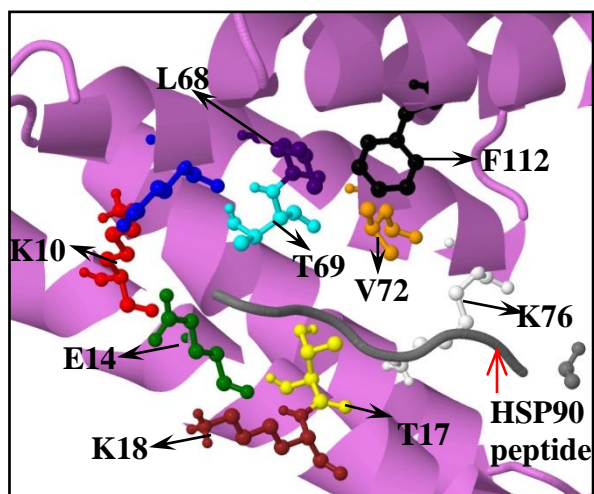

**C**

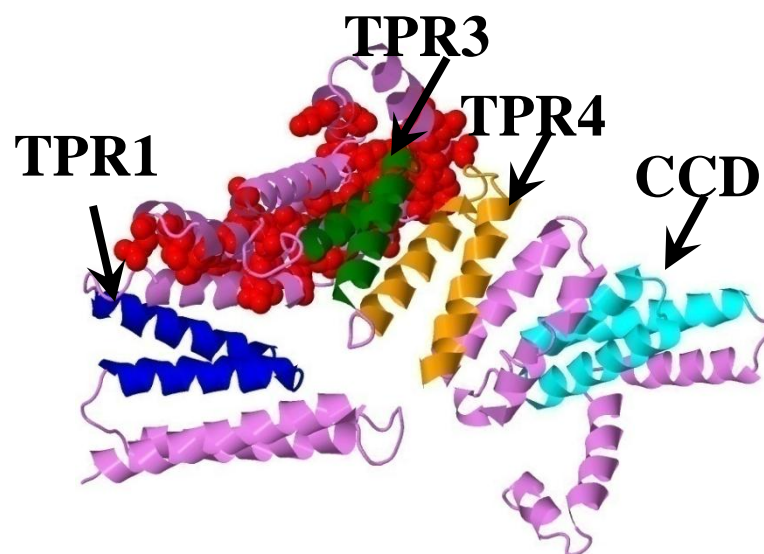

**D**

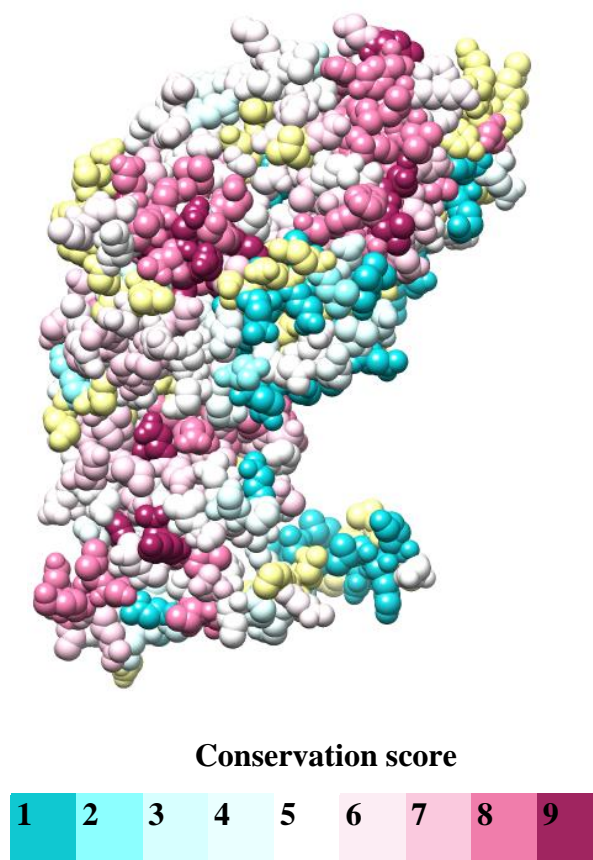

Supplement: Additional file 11 — A- Nrp1 predicted structure displayed in a surface representation complex with HSP90 peptide. Functional annotation was carried out using the COFACTOR server (Predicted GO terms for molecular function and biological process are: GO: 0022892 and GO: 0006606, respectively). The interaction was predicted using the structure of the designed TPR module complex with HSP90 (PDB ID: 3KD7) as reported by Cortajarena et al. [117].The arrow indicates the position of the peptide. B- Detailed view of HSP90 peptide interaction with Nrp1 residues. HSP90 peptide is shown in grey whereas the predicted interacting residues are shown in stick representation. C- Predicted structural model of Nrp1 shown in ribbon representation. Predicted TPRs 1, 3 and 4 are highlighted in different colors whereas acidic residues interrupting TPR2 are indicated in CPK models. The position of a coiled-coiled domain (CCD) predicted by SMART is 150–189 and is not shown. The position of a second predicted CCD found towards the C-terminus is highlighted. D- Space-filling representation of Nrp1 structure indicating the degree of conservation as calculated by the program ConSurf [118] based on Nrp1 amino acid sequence aligned against 52 NASP proteins from different organisms using MUSCLE. The color key is provided. [file 1471-2148-14-139-S11.pdf]
