# Supplementary material for: Molecular evolution of NASP and conserved histone H3/H4 transport pathway
Source: BMC Evol Biol. 2014 Jun 20;14:139. doi: 10.1186/1471-2148-14-139 (PMC4082323; doi:10.1186/1471-2148-14-139)
Supplement: Additional file 8: Figure S8 — A. Phylogenetic relationship among different fish-specific NASP paralogs. Tree topology corresponds to the ML method under the JTT+G model as predicted by MEGA. Branch lengths do not reflect genetic distance. Alternatively, the same topology was also recovered by the neighbour joining method using p-distances. Confidence values for ML and NJ trees are based on 1000 bootstrap replicates and are indicated (≥50%) in light-face and boldface (underlined), respectively. The tree is rooted with Halocynthia roretzi and Ciona intestinalis. B-C: %GC3 content and ENC values are shown in chart diagrams for the fish-specific NASP1 and NASP2 neighbouring genes, respectively. The black line separates GC3 and ENC whereas blue and red colors represent different neighbouring genes as indicated in the chart legends. D-G. Predicted fish-specific NASP1/2 TPRs 1–4 sequence alignments are shown in figures B to E, respectively. Reconstructed ancestral sequence using the ML method is shown in black background whereas different amino acid substitutions in each TPR motif for two paralogous proteins are shown in blue. H-K. Predicted NASP1/2 TPRs 1–4 sequence alignments are shown in figures H to K, respectively, for various lineages. [file 1471-2148-14-139-S8.pdf]

Figure S8A: Phylogenetic relationship among different fish specific NASP paralogs. Tree topology corresponds to the ML method under the JTT+G model as predicted by MEGA. Branch lengths do not reflect genetic distance. Alternatively, the same topology was also recovered by the neighbour joining method using p-distances. Confidence values for ML and NJ trees are based on 1000 bootstrap replicates and are indicated (≥ 50%) in light-face and boldface (underlined), respectively. The tree is rooted with *Halocynthia roretzi* and *Ciona intestinalis.* **Figure S8, B-C:** %GC3 content and ENC values are shown in chart diagrams for the fish-specific NASP1 and NASP2 neighbouring genes, respectively. Black line separates GC3 and ENC whereas blue and red colors represent different neighbouring genes as indicated in the chart legends. **Figure S8, D-G:** Predicted fish specific NASP1/2 TPRs 1-4 sequence alignments are shown in figures B to E, respectively. Reconstructed ancestral sequence using the ML method is shown in black background whereas different amino acid substitutions in each TPR motif for two paralogous proteins are shown in blue. **Figure S8, H-K:** Predicted NASP1/2 TPRs 1-4 sequence alignments are shown in figures H to K, respectively, for various lineages.

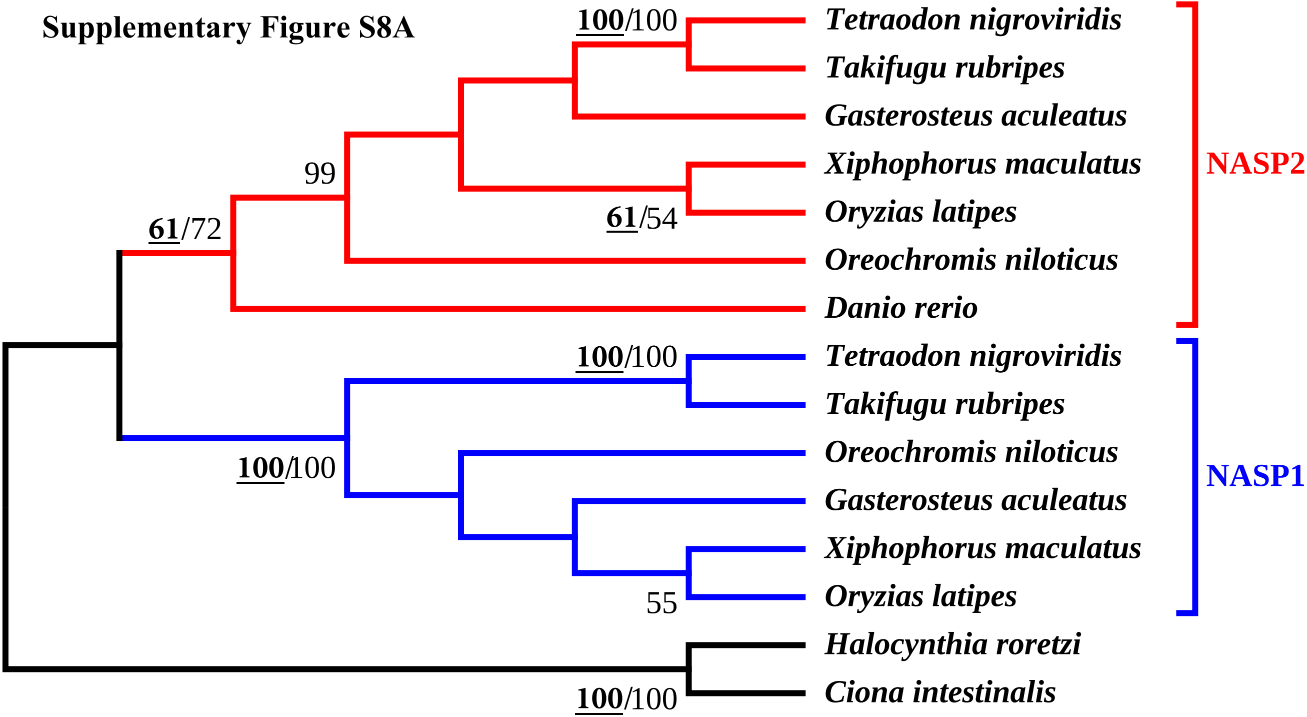
Supplementary Figure S8A
100/100
Tetraodon nigroviridis
Takifugu rubripes
Gasterosteus aculeatus
99
Xiphophorus maculatus
Oryzias latipes
61/54
61/72
Oreochromis niloticus
Danio rerio
NASP2
100/100
Tetraodon nigroviridis
Takifugu rubripes
Oreochromis niloticus
100/100
Gasterosteus aculeatus
Xiphophorus maculatus
Oryzias latipes
55
NASP1
Halocynthia roretzi
Ciona intestinalis
100/100

B

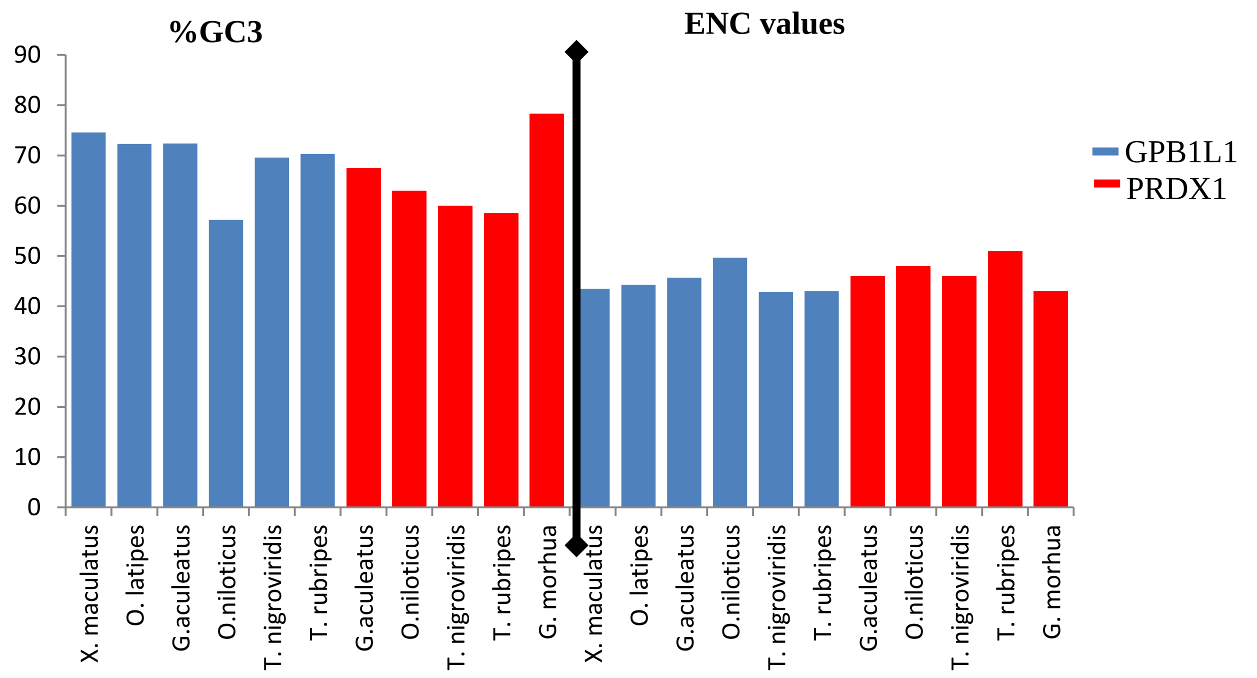
%GC3
ENC values
GPB1L1
PRDX1
90
80
70
60
50
40
30
20
10
0
X. maculatus
O. latipes
G.aculeatus
O.niloticus
T. nigroviridis
T. rubripes
G.aculeatus
O.niloticus
T. nigroviridis
T. rubripes
G. morhua
X. maculatus
O. latipes
G.aculeatus
O.niloticus
T. nigroviridis
T. rubripes
G.aculeatus
O.niloticus
T. nigroviridis
T. rubripes
G. morhua

C

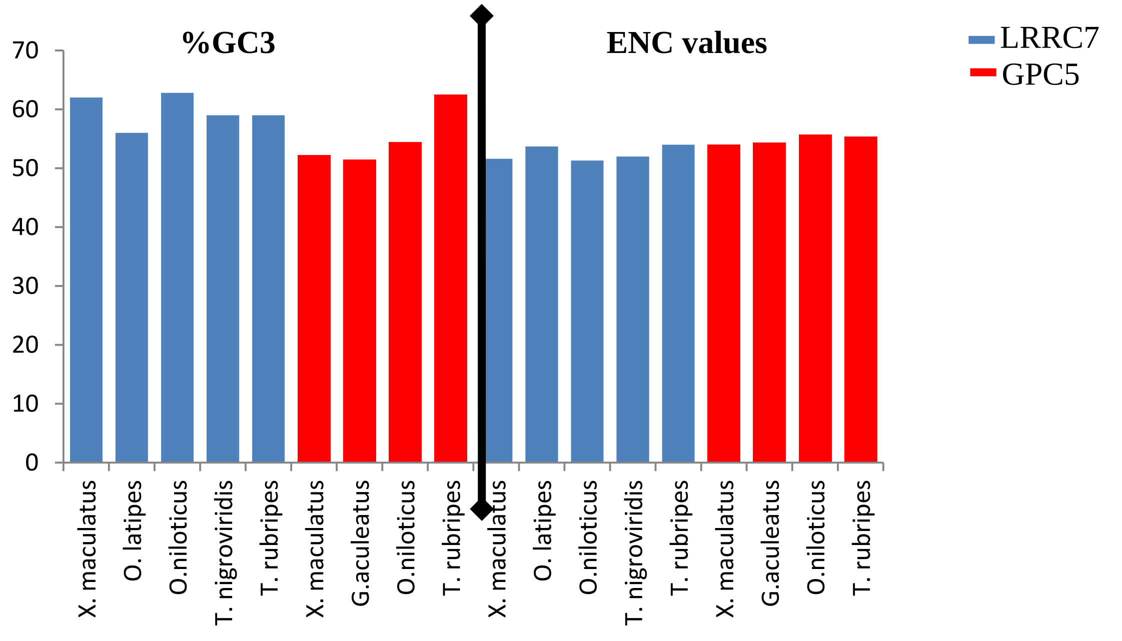
%GC3
ENC values
LRRC7
GPC5
70
60
50
40
30
20
10
0
X. maculatus
O. latipes
O.niloticus
T. nigroviridis
T. rubripes
X. maculatus
G.aculeatus
O.niloticus
T. rubripes
X. maculatus
O. latipes
O.niloticus
T. nigroviridis
T. rubripes
X. maculatus
G.aculeatus
O.niloticus
T. rubripes

D

X.maculatus/1-34	AKKLIGTGKRHLVMGDVVSAVNVFQEACSMLAAK
O.latipes/1-34	AKKLIGTGKRHLVMGDVVSAVNVFQDACSILASK
G.aculeatus/1-34	ANKLIGTGNRHLVMGDVVSAVRVFQDACSMLAAR
O.niloticus/1-34	AKKLIGTGKRHLVMGDVVSAVSVFQEACGMLAEK
T.nigroviridis/1-34	AKKLVGAGNKYLVLGDVVSAVGVFQDACSMLAAK
T.rubripes/1-34	AKKLVGAGNKYLVLGDVVSAVAVFQDACSMLAAK
D.rerio/1-34	AKKLIGTGSRHLVMGDVVSAVSVFQEACAMLAEK
Ancestral/1-34	AKKL?G?GKKHLVMGDV?SAVNAFQEACS?LAEK
X.maculatus/1-34	ANKLVGTGKKFLVTGKVVEAVSALQEACGMLAKK
O.latipes/1-34	ANKLIGAGKKFLVMGKAVEAVSALQEACGMLAKQ
G.aculeatus/1-34	ANKLIGSGKKHLVMGKVVEAVSALQEACGMLAQK
O.niloticus/1-34	ANKLIGTGKKHLVMGKVVEAVSTLQEACGMLAKK
T.nigroviridis/1-34	ANKLVGAGKKHLVMGKVVEAVNSLQEACGILAKT
T.rubripes/1-34	ANKLIGAGKKHLVMGKVVEAVNSLQEACGMLAKT

NASP-2

E

X.maculatus/1-34	GEAFFLCGKSLLELAR---LQLAWEMLEVAKVIFKRK
O.latipes/1-34	GEAVFLYGKSLLELAR---LQLAWEMLEVAKLIYKRK
G.aculeatus/1-34	AEAFFLCGKSLLELAR---LQLAWEMLEVAKVIYKRK
O.niloticus/1-34	GEAFFFCGKSLLELAR---LQLAWEMLEVAKVIYKRK
T.nigroviridis/1-34	GEALFLCGKSLLELAR---LQLAWEMLEVAKVIYKRK
T.rubripes/1-34	GEALFLCGKSLLELAR---LQLAWEMLEVAKVIYKRK
D.rerio/1-34	GEVFFFCGKALLELAR---LRLAWEMLEVAKVIYKRK
Ancestral/1-34	GEAFFF YGKSLLELAR---LQLAWEML?LAK?I?LR?
X.maculatus/1-34	GEAFYWCGKALLDLAR---LQLAWEMLEVAKVIYRRK
O.latipes/1-34	GEAFLWCGKALLDLAR---LQLAWEMLEVAKSIYKRK
G.aculeatus/1-34	GEAFFWCGKALLDLAR---LQLAWEMLEVAKVIYKRK
O.niloticus/1-34	GEAFFWYGKALLDLAR---LQLAWEMLEVAKVIYKRK
T.nigroviridis/1-34	GEAFFWCGKALLDLAR---LQLAWEMLEVAKVIYKRK
T.rubripes/1-34	GEAFFWCGKALLDLAR---LQLAWEMLEVAKVIYKRK

NASP-2

F

X.maculatus/1-34	AQAYLKLGEVSAETGNYPQALEDFQECLIIQLKL	NASP-2
O.latipes/1-34	AQAHLKLGEVGAETGNYPQALEDFQECLAIQLQH	
G.aculeatus/1-34	AQAYLKLGEVSAESGNYSQALEDFQECLALQLKH	
O.niloticus/1-34	AQAYLKLGEVSAESGNYPQALDDFQECLALQLKH	
T.nigroviridis/1-34	AQIYLKLGEVSAESGNYPQALEDFQECLCLQLKY	
T.rubripes/1-34	AQIYLKLGEVSAESGNYPQALEDFQECLCLQLKH	
D.rerio/1-34	AQIHLKLAEVGVESGNYSQALEDSNECLTLQLKH	
Ancestral/1-34	AQAHLKLGEVSVESGNYPQA?EDFQ?CL?IQK?H	
X.maculatus/1-34	AQTHLKLGEVSAESGNYNQALEDFQECLKVQLKH	
O.latipes/1-34	AQAHLKLGEVSAESGNYTQALEDFQECLKLQVKH	
G.aculeatus/1-34	AQAHSKLGEVSSESGNYTQALEDFQECLKLQVKH	
O.niloticus/1-34	AQAHLKLGEVSAESGNYPQALEDFQECLKLQVKH	
T.nigroviridis/1-33	AQAHLKLGEVSVESGNYTQALDDFQECLKLQAK-	
T.rubripes/1-34	AQAHLKLGEVSVESGNYTQALDDFEECLKLQLKH	

G

X.maculatus/1-34	AETHYHVATTLVFMDQYDQAIKHYNSSVKVIETR	NASP-2
O.latipes/1-34	AETHYHVATTLCYMDEYRQAIQHYNSSIEVIENR	
G.aculeatus/1-34	AETHYHVATTLCYMDQYSQAIQHYNSSIKVIETR	
O.niloticus/1-34	AETHYHVATTLCYMDQYSQAIQHYNSSIKVIETR	
T.nigroviridis/1-34	AETHYHVATTLCYMDKYSQAIQHYNSSIEVIEKR	
T.rubripes/1-34	AETHYHVATTLCYMDKYSQAIQHYNSSIEVIEKR	
D.rerio/1-34	TETHYQLGTTYSYTTQYNQAIEHFSNSIKVIESR	
Ancestral/1-34	AETHYQLGLAYSYN?QYD?AI?HFN?SI?VIE?R	
X.maculatus/1-34	AETHYQLGVTHSLNLQYVPAIQALNNSISVIKNR	
O.latipes/1-34	AETHYQLGLTYSLNLQYSPAIEALNNSISVIKSR	
G.aculeatus/1-34	AETHYQLGLTYSLDVQYGRAIEELKSSISVIKSR	
O.niloticus/1-34	AETHYQLGVTYSLNTQYSEAIESLKSSISIIKNR	
T.nigroviridis/1-34	AETHYQLGLTHGLNLQHGAAVAELSRSISVIKSR	
T.rubripes/1-34	AETHYQLGLTYGLNLQYNQAIAELNRSISVIKSR	

H

C.elegans-NASP1	LAELLAAGRRALKVNDIDKASDSLSEATELSSEI
C.elegans-NASP2	LSENIIAGRRFMRTDVFDKAVDVLSVAASIAAEV
C.briggsae-NASP1	AAELLAAGRRALKVSDYETATEALSEASEMIVEL
C.briggsae-NASP2	YTEQLIAGRRFMKTNVFDKATEALSLAAALGAEI
*I.tridecemlineatus*_NASP1	AKRLLGLGQKHLVMGDIPAAVNAFQEAASLLGKK
*I.tridecemlineatus*_NASP2	VKKLLGLGQKHLVVGDIPVA----EEAASLLGKR
*G.gorilla*_tNASP	AKKLLGLGQKHLVMGDIPAAVNAFQEAASLLGKK
*G.gorilla*_NASP2	----------------------------------

I

C.elegans-NASP1	FDSLYYYGMATLELAK--MKLSWEILETARCIAAAK
C.elegans-NASP2	FEANFLYGKALLEVGK--MKLAWELLETSRCIADKK
C.briggsae-NASP1	YEYYYYYGMATLELGK--MKLAWEVLENARCIAMAK
C.briggsae-NASP2	FDANFLYGKALLELSK--IKLAWELLETSRCICEKK
*I.tridecemlineatus*_NASP1	GEAFFFYGKSLLELAR--LELAWDMLDLAKIIFKRQ
*I.tridecemlineatus*_NASP2	GEDFFFYGKSQL------LEL-LDILDLAKIIFKSQ
*G.gorilla*_tNASP	GEAFFFYGKSLLELAR--LELAWDMLDLAKIIFKRQ
*G.gorilla*_NASP2	------------------LELAWNTLDLAKIVFKRQ

J

C.elegans-NASP1	ADVLVLLGEHGISDGKYTQAFEDLDRALNIQRNV
C.elegans-NASP2	ADVLTSLGEHGIADSKYEQAQKDLTEAISIQTVH
C.briggsae-NASP1	ADVLVVLGDHSVSDGKYDQALEDLEQALEIQKNV
C.briggsae-NASP2	ADVLTSLGEHGIVDSKYEQAQKDLAEAVAIQSAL
*I.tridecemlineatus*_NASP1	AQAHLKLGEVSVESENYIQAVEEFQACLNLQEQY
*I.tridecemlineatus*_NASP2	---HIKLGEVYVESENYIQAMEVFQTFLNLQEQC
*G.gorilla*_tNASP	AQAHLKLGEVSVESENYVQAVEEFQSCLNLQEQY
*G.gorilla*_NASP2	AQARLKLGEVGVESENYVQAVEEFQSCLKLWEQY

K

C.elegans-NASP1	AQTYILIGNACASDANYDETVQYFGKTKDVLIAR
C.elegans-NASP2	ANTTHLLAKAFSSDSLFEQAAAHFNDTKNILIAK
C.briggsae-NASP1	AQTYILMADACTSGMNYDEAINYFEKTKETLKSR
C.briggsae-NASP2	ANTTHLLAKSFSLDSIFEKAATHFCESKNILIAK
*I.tridecemlineatus*_NASP1	AETHYQLGLAYGYNSQYDEAVAQFNKSIEVIEKR
*I.tridecemlineatus*_NASP2	AETHYQL-FAYGYNSQCDEAVAQFNRSIEALEKR
*G.gorilla*_tNASP	AETHYQLGLAYGYNSQYDEAVAQFSKSIEVIENR
*G.gorilla*_NASP2	AETHYQLGLAYGYNSQYDEAVAQFNKSIEVIEKR
