# Supplementary material for: Molecular evolution of NASP and conserved histone H3/H4 transport pathway
Source: BMC Evol Biol. 2014 Jun 20;14:139. doi: 10.1186/1471-2148-14-139 (PMC4082323; doi:10.1186/1471-2148-14-139)
Supplement: Additional file 12 — Supplementary methods for structural prediction and calculation of electrostatic interaction properties. [file 1471-2148-14-139-S12.pdf]

Supplemental Methods

Structural bioinformatics and functional annotation of Nrp1:

The three dimensional structure of proteins (see supplementary figure 7 for lineages used) were predicted using I-TASSER server which is a hierarchical protein structure modeling approach based on the secondary-structure enhanced Profile-Profile threading Alignment

(PPA) [1]. Sequence-to-structure-to-function paradigm algorithm is used to predict the protein structure from the primary amino acid sequences. The reliability of the structure is reported as confidence score (C-score) which ranges from -5 to 2 with higher value signifying the more reliable prediction. C-score is calculated based on the significance of threading template alignments and the convergence parameters of the structure assembly simulations [1, 2]. The predicted structures were visualized using Jmol software. For functional annotation, Nrp1 predicted structure was submitted to COFACTOR which is a structure based method for biological function annotation. It starts with the identification of template proteins with similar fold by searching the template libraries carrying the known protein–ligand binding interactions, Enzyme Commission numbers and Gene Ontology terms. Using the identified functional templates insights into the biological function of the query structure are deduced with the confidence score based on the scoring function that combines both global and local structural similarities [3].

Analysis of electrostatic potentials

NASP family proteins contain stretches of acidic residue and have an overall negative charge. It suggests that electrostatic interaction properties might have a potential selective role in NASP evolution. Because crystal structure is not available for any of NASP proteins, therefore, we used I-TASSER server to predict the structure of proteins listed in supplementary figure 7 and submitted the resulting structures on web-PIPSA pipeline [4]. The electrostatic potentials were determined using University of Houston Brownian Dynamics program [5] and their corresponding similarity indices were also computed.

References:

1. Zhang Y: **I-TASSER server for protein 3D structure prediction**. *BMC bioinformatics* 2008, **9**:40.
2. Roy A, Kucukural A, Zhang Y: **I-TASSER: a unified platform for automated protein structure and function prediction**. *Nature protocols* 2010, **5**(4):725-738.
3. Roy A, Yang J, Zhang Y: **COFACTOR: an accurate comparative algorithm for structure-based protein function annotation**. *Nucleic acids research* 2012, **40**(Web Server issue):W471-477.
4. Richter S, Wenzel A, Stein M, Gabdoulline RR, Wade RC: **webPIPSA: a web server for the comparison of protein interaction properties**. *Nucleic acids research* 2008, **36**(Web Server issue):W276-280.
5. Madura JD, Briggs JM, Wade RC, Davis ME, Luty BA, Ilin A, Antosiewicz J, Gilson MK, Bagheri B, Scott LR *et al*: **Electrostatics and diffusion of molecules in solution: simulations with the University of Houston Brownian Dynamics program**. *Computer Physics Communications* 1995, **91**(1–3):57-95.
